# Nanoconfinement‐Induced Electrochemical Ion‐Solvent Cointercalation in Pillared Titanate Host Materials

**DOI:** 10.1002/anie.202423593

**Published:** 2025-03-11

**Authors:** Mennatalla Elmanzalawy, Haohong Song, Maciej Tobis, Robert Leiter, Jaehoon Choi, Hyein Moon, Wan‐Yu Tsai, De‐en Jiang, Simon Fleischmann

**Affiliations:** ^1^ Helmholtz Institute Ulm (HIU) 89081 Ulm Germany; ^2^ Karlsruhe Institute of Technology (KIT) 76021 Karlsruhe Germany; ^3^ Interdisciplinary Materials Science Vanderbilt University, Nashville Tennessee 37235 United States; ^4^ Univ. Lille, CNRS, Univ. Polytechnique Hauts-de-France, UMR 8520 - IEMN F-59000 Lille France; ^5^ Réseau sur le Stockage Electrochimique de l'Energie (RS2E), CNRS FR 3459, 33 rue Saint Leu, Amiens Cedex 80039 France; ^6^ Department of Chemical and Biomolecular Engineering, Vanderbilt University, Nashville Tennessee 37235 United States

**Keywords:** Energy storage, intercalation, layered compounds, nanoconfinement

## Abstract

Electrochemical ion‐solvent cointercalation reactions are an avenue to reach improved kinetics compared to the corresponding intercalation of desolvated ions. Here, we demonstrate the impact of different structural pillar molecules on the electrochemical Li^+^ intercalation mechanism in expanded hydrogen titanate (HTO) electrode materials. We show that interlayer‐expansion of HTO with organic pillars can enable cointercalation reactions. Their electrochemical reversibility is drastically improved when non‐cross‐linking pillars are employed that expand and separate the host material's individual layers, underlining the impact of the electrochemo‐mechanics of the nanoconfined interlayer space. This pillared HTO structure results in an increased Li^+^ storage capacity and reversibility compared to pristine HTO. We derive structural models of the pillared HTO host materials based on combined experiments and theoretical calculations, and employ electrochemical operando experiments to unambiguously demonstrate the nanoconfinement‐induced cointercalation mechanism in pillared HTO electrode materials. The work demonstrates the potential of nanoconfined pillar molecules to modify host materials and enable highly reversible cointercalation reactions with improved capacity and kinetics.

## Graphical abstract

Interlayer‐expansion of hydrogen tetratitanate is realized with organic pillars. Optimized atomistic configurations of the pillared materials are proposed by characterization with experiment and simulation. The nanoconfinement modification of HTO induces ion‐solvent cointercalation reactions with improved kinetics and increased storage capacity.

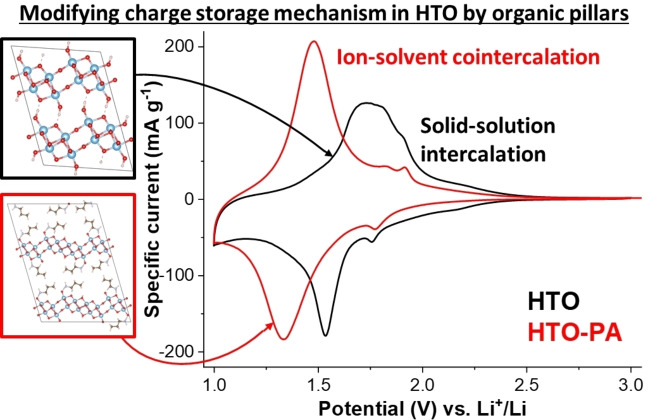

Received: 29.December 2024

## 1. Introduction

Electrochemical energy storage devices, including lithium‐ion and sodium‐ion batteries, are in increasing global demand. Their functioning mechanism is the electrochemical intercalation of ions from the electrolyte into the solid‐state host structure of the electrode materials. Layered transition metal oxides (TMOs) are widely used as cathode materials and serve as alternatives to carbon‐based anodes in lithium‐ and sodium‐ion batteries. Among them, titanate‐based materials have attracted interest due to their relative abundance, low toxicity and structural/chemical tunability. They can often serve as host structures for both lithium‐ and sodium‐ions making them highly versatile.[^1^, ^2^, ^3^]

Hydrogen titanates are a family of metastable materials derived from layered alkali titanates.[^4^, ^5^] They are typically synthesized in acidic solution by etching and topotactic exchange of the alkali ion in the interlayer space by protons and/or water.[^5^, ^6^, ^7^] This provides them with a high degree of structural/chemical tunability of their nanoconfined interlayer composition, comparable to two‐dimensional Ti_3_C_2_T_x_ MXenes derived via a similar selective etching approach.[^8^, ^9^] These materials’ nanoconfinement environment, in turn, can have an effect on their electrochemical ion intercalation properties.[^10^, ^11^, ^12^, ^13^]

In the layered hydrogen titanate H_2_Ti_3_O_7_, it was found that confined interlayer protons are the determining structural feature to enable electrochemical H^+^ intercalation.^[14]^ This is because they separate the titanate layers, allowing to effectively compensate electrochemically induced strain by contraction/expansion without altering intralayer structure.^[14]^ Interlayer‐expansion of layered titanate hollow spheres by confined Mg^2+^ ions was demonstrated as a design strategy for electrochemical Na^+^ intercalation hosts.^[15]^ An investigation into different types of confined alkali ions (Li^+^, Na^+^, K^+^, Cs^+^) within lepidocrocite‐type layered titanates revealed that the interlayer spacing is expanded with increasing ion size (0.68 to 0.85 nm).^[16]^ However, sodium‐modified titanate showed the highest capacity for electrochemical Na^+^ intercalation hosts (153 mAh g^−1^), even though this material has a smaller interlayer spacing than the cesium‐modified titanate (95 mAh g^−1^), demonstrating the importance of nanoconfinement chemistry in addition to just geometrical considerations.^[16]^ In the layered hydrogen titanate H_2_Ti_2_O_5_ system, it was found that K^+^ intercalation can be accomplished most efficiently, especially at low temperatures (−40 °C), for the material with confined interlayer water (H_2_Ti_2_O_5_⋅H_2_O, “HTO”), compared to its dehydrated form with additional interlayer defects (“QTO”) that also exhibits smaller interlayer spacing (0.78 nm for QTO vs. 0.84 nm for HTO).^[17]^ The authors attribute this to ion‐solvent cointercalation from the organic electrolyte into HTO, which is enabled by strong K^+^/1,2‐dimethoxyethane binding energy (electrolyte effect) and a charge shielding effect of confined interlayer H_2_O (electrode effect).^[17]^ The emergence of such cointercalation phenomena is gaining more and more interest, as it offers the opportunity to substantially modifying electrode reactions regarding redox potential, capacity and kinetics and even enable reactions not feasible for desolvated ions.[^18^, ^19^, ^20^, ^21^] However, the phenomenon is almost exclusively studied as an electrolyte effect, while the influence of the electrode on cointercalation is widely neglected.^[22]^

This motivates us to explore the structural motifs that enable such favorable electrochemical cointercalation effects in titanates. Besides interlayer modification of layered titanates with simple cations or crystal water, the introduction of organic pillar molecules like linear alkyl (di)amines offers an even wider materials design space,[^23^, ^24^, ^25^, ^26^, ^27^] with the potential to induce effective cointercalation phenomena.^[22]^ However, no clear materials design rules for host materials on how to control cointercalation have emerged so far. Specifically, there is an urgent need to identify how electrochemically induced strain is compensated in strongly expanded/pillared titanates and whether layer‐separating or cross‐linking pillaring approaches favor electrochemical kinetics.

In this work, we investigate the layered titanate H_2_Ti_4_O_9_⋅H_2_O and increase its interlayer spacing from 0.88 to 1.58 nm by insertion of organic molecular pillars. This first step enables the study of nanoconfinement geometry‘s influence on electrochemical ion intercalation. Furthermore, using either propylamine (PA) or 1,6‐hexanediamine (HDA) pillars, an identical expanded interlayer spacing is obtained. But while HDA pillars with two ammonium groups cross‐link the titanate layers, PA pillars are non‐cross‐linking, likely impacting how electrochemically‐induced strain is compensated within the host lattice. We utilize a combination of experimental and theoretical methods to derive precise structural models of the pillared titanates. Electrochemical Li^+^ intercalation capacity from organic electrolyte can be increased from ca. 2.1 to 2.95 Li^+^ per tetratitanate when pillaring with non‐cross‐linking PA molecules. Using a combination of operando X‐ray diffraction (XRD), electrochemical dilatometry (ECD) and electrochemical quartz crystal microbalance (EQCM), we unambiguously identify a nanoconfinement‐induced change in intercalation mechanism from solid‐solution intercalation to ion‐solvent cointercalation.

## 2. Results and Discussion

### 2.1. Physicochemical Characterization

**Figure 1:**
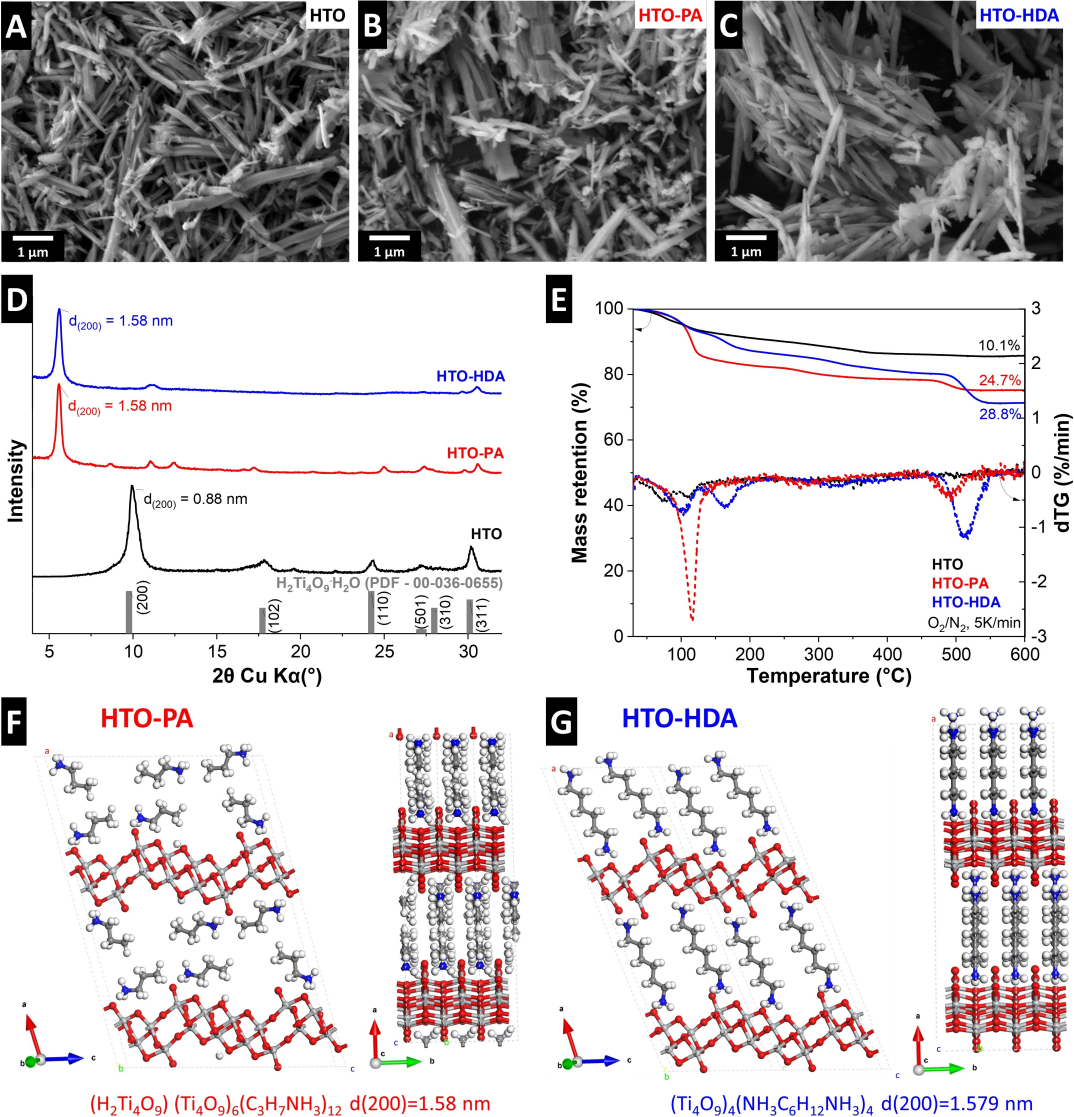
Structure and composition of HTOs: Scanning electron microscopy images of (A) H_2_Ti_4_O_9_⋅H_2_O (HTO), (B) HTO‐HDA and (C) HTO‐PA. (D) Powder XRD patterns of HTO, HTO‐PA, HTO‐HDA, and the PDF of H_2_Ti_4_O_9_⋅H_2_O as reported in the literature. (E) TGA and dTG (derivative thermogravimetry) plots for HTO, HTO‐PA and HTO‐HDA, showing mass loss steps and quantities. Optimized crystal structures of (F) HTO‐PA and (G) HTO‐HDA, obtained from DFT calculations and considering structural formulae derived from TGA (legend for atom assignment: light grey: Ti, red: O, white: H, blue: N, dark grey: C).

Layered potassium tetratitanate is synthesized via a solid‐state synthesis of TiO_2_ and K_2_CO_3_ at 800 °C, yielding monoclinic K_2_Ti_4_O_9_ (KTO) with a rod‐like morphology (Figure S1). The confined interlayer potassium is subsequently exchanged by protons and water via treatment in HCl according to earlier work of Izawa et al..^[6]^ The resulting hydrogen tetratitanate H_2_Ti_4_O_9_⋅H_2_O (HTO) retains the rod‐like morphology of KTO as indicated by scanning electron microscopy (SEM, Figure 1A). The rods measure up to several microns in lengths and several hundreds of nanometers in diameter, in agreement with previous reports.^[28]^ The chemical composition of the nanoconfined interlayer environment of HTO can be further modified with organic amine‐based molecular pillars replacing interlayer water^[23]^ without leading to any visible modification of the macroscopic rod‐like morphology as shown by SEM micrographs (Figure 1B–C). Propylamine (PA) and 1,6‐hexanediamine (HDA) are selected as molecular pillars with one or two anchoring amine groups, respectively. The reason is the assumption that monofunctional PA bonds to one layer of the titanate host resulting in a separated HTO structure, while bifunctional HDA interacts with two adjacent titanate layers leading to a cross‐linked HTO structure. Hence the pillar choice allows to compare the influence of interconnectivity of the pillared structure on the volumetric expansion behavior when used as electrochemical ion intercalation hosts.

To examine the pillar‐induced changes in crystal structure, powder X‐ray diffraction (XRD) patterns of the pristine HTO and the molecularly pillared HTO‐PA and HTO‐HDA are shown in Figure 1D. The XRD pattern of as‐synthesized HTO is in close agreement with that reported in literature (PDF 00–036‐0655), which indicates the successful synthesis of the protonated/hydrated phase from the precursor K_2_Ti_4_O_9_. ICP results (Table S1) show that only negligible amounts of potassium remain in the structure (ca. 0.2 wt.%), indicating successful ion exchange by acid treatment. The introduction of molecular pillars causes a significant shift in the (200) plane peak to lower angles, indicative of an increase in the interlayer spacing from 0.88 nm in the pristine HTO to 1.58 nm in both HTO‐PA and HTO‐HDA. The similar interlayer spacing and morphology of the two molecularly pillared titanates, HTO‐PA and HTO‐HDA, permits to exclusively assess the impact of cross‐linking versus layer‐separating/non‐cross‐linking molecular pillars on the electrochemical properties.

Quantification of nanoconfined interlayer molecules (water, or organic pillars) is conducted using thermogravimetric analysis (TGA, Figure 1E). Heating to 600 °C in synthetic air atmosphere converts H_2_Ti_4_O_9_ into 4 TiO_2_ by dehydration/dehydroxylation[^29^, ^30^] (corresponding to 94.7 % of the initial mass). At the same time, there is a complete removal of nanoconfined molecular species by combustion and/or evaporation, hence any additional mass loss can be attributed to interlayer molecules.^[14]^ For pristine HTO, an overall mass loss of 10.1 % is measured, indicating an initial composition with a structural formula H_2_Ti_4_O_9_ ⋅ 0.99H_2_O. The measured overall mass loss for HTO‐PA is 24.7 %, indicating the initial composition H_2_Ti_4_O_9_ ⋅ 1.47PA, and for HTO‐HDA it is 28.8 % suggesting the structural formula H_2_Ti_4_O_9_ ⋅ 0.96HDA. Hence it is reasonable to approximate the nanoconfined interlayer chemistry of HTO, HTO‐PA and HTO‐HDA as 1 H_2_O, 1.5 PA and 1 HDA molecules, respectively. These values are further utilized for theoretical calculation of the structure.

Taking into account the measured interlayer spacing of HTO‐PA and HTO‐HDA, as well as the quantification of nanoconfined pillaring molecules, the energetically favorable pillar conformation within the interlayer space is calculated using density functional theory (DFT, Figure 1F–G). For the HTO‐PA system, we considered three configurations: fully perpendicular, fully parallel, and mixed models with respect to the HTO basal plane. The calculations show that in the fully perpendicular configuration, the d‐spacing is only 1.38 nm, which is too short in comparison with the experimental value of 1.58 nm. As more PA molecules are aligned parallel to the basal plane, the d‐spacing gradually increases. The PA bilayer structure allows the PA molecules to interact more effectively with the OH groups on both the upper and lower HTO layers. In the lowest energy configuration, this leads to proton transfer from HTO to PA involving the formation of propylammonium and resulting in a d‐spacing of 1.58 nm (Figure 1F). For the HTO‐HDA system, we considered two scenarios: HDA aligned parallel or perpendicular to the HTO basal plane. DFT structural optimization revealed that in the perpendicular configuration, both NH_2_ groups of HDA are protonated by the top and bottom HTO layers to form hexanediammonium in the lowest energy configuration (Figure 1G). In contrast, when HDA is aligned parallel to the basal plane, the energy is higher due to lack of ionic interaction and the d‐spacing is shorter at 1.51 nm, in comparison with the experimental value.

**Figure 2:**
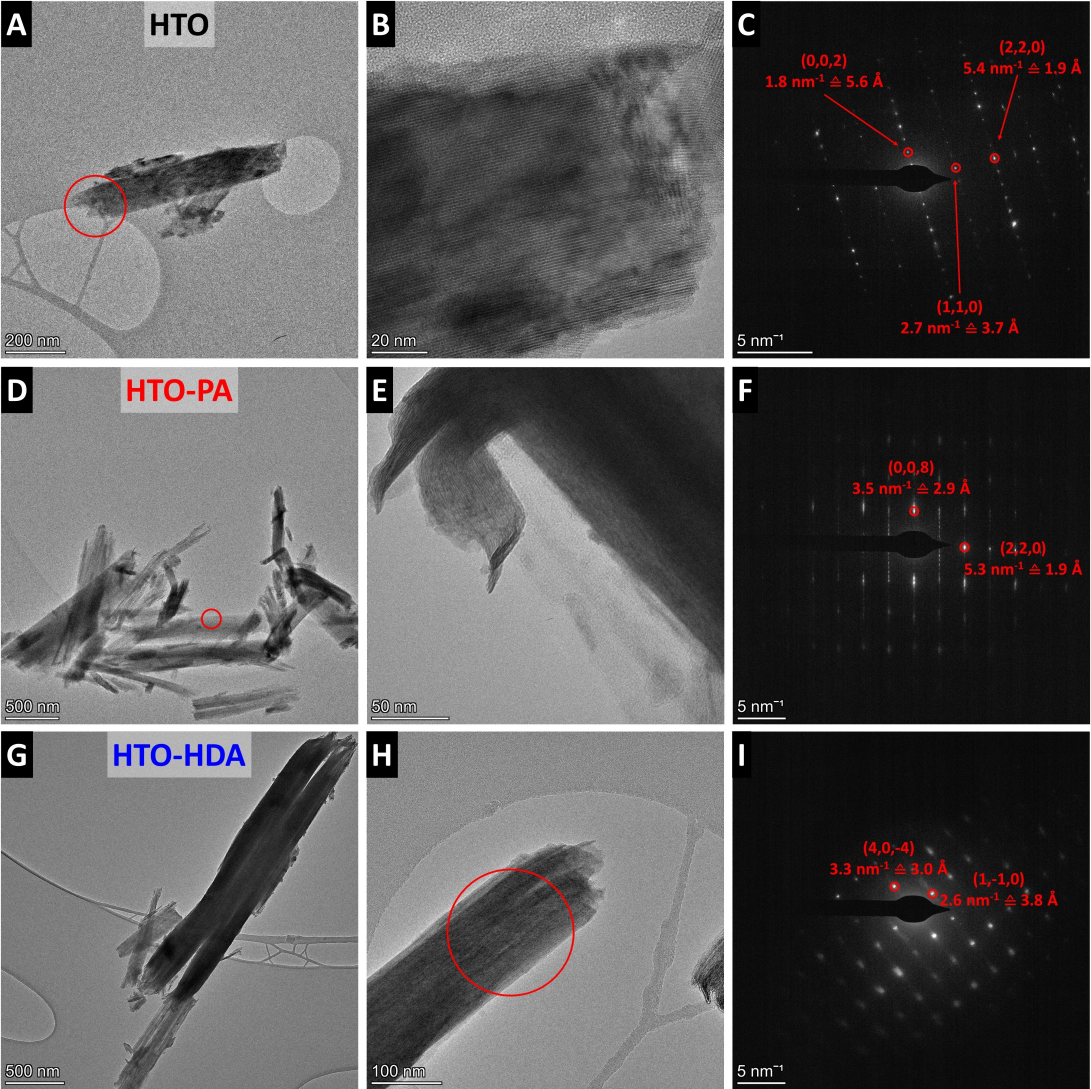
Transmission electron microscopy images and selected area electron diffraction pattern (area indicated by red circle) of (A−C) HTO, (D−F) HTO‐PA, and (G−I) HTO‐HDA.

Analysis of the structural properties of HTOs with different nanoconfinement chemistries at a nanoscale is conducted using transmission electron microscopy (TEM, Figure 2). All samples (HTO, HTO‐PA and HTO‐HDA) demonstrate rod‐like morphology (Figure 2A‐B, 2D‐E, 2G‐H), confirming prior SEM results. This allows to further analyze free‐standing rods individually using selected area electron diffraction (SAED). The recorded patterns for single crystals are then compared to the DFT‐optimized crystal structures to verify the proposed structural models. For the pristine HTO sample, the SAED pattern (Figure 2C) aligns well with the simulated H_2_Ti_4_O_9_ structure (see supporting information for the coordinates). Specifically, the (002) reflection yields a d‐spacing of 0.56 nm, which closely matches the 0.57 nm obtained from the calculated structure. Similarly, the (110) and (220) reflections, with d‐spacings of 0.37 nm and 0.19 nm, respectively, correspond precisely to the values derived from the calculated pattern (0.37 nm and 0.19 nm).

For the HTO‐PA sample, the SAED‐measured (008) reflection with d‐spacing measuring 0.29 nm was comparable to the simulated value of 0.287 nm, and the (220) reflection at 0.19 nm also agreed with the predicted value. Similarly, for the HTO‐HDA sample, the SAED‐derived (40‐4) reflection with d‐spacing of 0.30 nm and (1‐10) with d‐spacing of 0.38 nm were consistent with the corresponding simulated values of 0.30 nm and 0.38 nm, respectively. Overall, the TEM and SAED analysis confirm the validity of the proposed structural models for the pillared tetratitanates. Additionally, calculated XRD pattern of the structural models of HTO‐PA and HTO‐HDA are given in Figure S2. It should be noted that the experimental structural analysis is limited to the titanate host, while the conformation of organic pillars is indirectly concluded based on minimized free energy and previous literature reports of confined alkyl(di)amine pillars.[^31^, ^32^, ^33^]

The interaction between nanoconfined pillars within the interlayer and the HTO‐based host lattice is analyzed by Raman spectroscopy. This allows to assess any organic pillaring‐induced changes in bonding environment. The presence of organic pillars in HTO‐PA and HTO‐HDA is confirmed by signals in the C−H stretching region (2800 to 3000 cm^−1^).^[34]^ Moreover, the spectra of HTO, HTO‐PA and HTO‐HDA are highly comparable in the wavenumber region below ca. 900 cm^−1^ typically assigned to the Ti−O lattice vibrations,^[35]^ with no new major signals appearing. Shifts or broadening of certain signals can provide information about host‐pillar interactions. In HTO, the band at 267 cm^−1^ attributed to the Ti‐OH bonds[^36^, ^37^] blue shifts to 275 cm^‐1^ after pillaring with either PA or HDA. The Raman signals of HTO at 394, 452, 681 and 866 cm^−1^ have previously been assigned to protonated tetratitanate.[^28^, ^38^] They are still present (394 and 452 cm^−1^), red shift (681 to ca. 661 cm^−1^) or broaden (866 cm^−1^) after organic pillaring with PA and HDA. Other peaks arising from the organic pillars are present in HTO‐PA and HTO‐HDA and can be attributed to different components of the organic molecules (Figure S3). The overall strong similarity of the spectra is indicative of the tetratitanate intralayer structure remaining intact after pillaring. Slight shifts/broadening of protonated titanate related signals suggests an interaction between the organic pillars and the terminal protons of the titanate layers.

**Figure 3:**
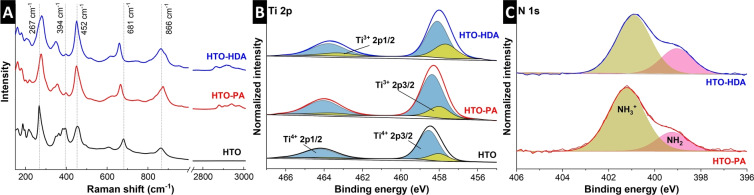
Analysis of host‐pillar interaction. (A) Raman spectra, and XPS of (B) Ti 2p and (c) N 1s regions of HTO, HTP‐PA, and HTO‐HDA samples.

The nature of this interaction is further elucidated using X‐ray photoelectron spectroscopy (XPS, Figure 3B–C). The Ti 2p region in pristine HTO and pillared HTO‐PA and HTO‐HDA all contain a mixture of Ti oxidation states Ti^4+^ and Ti^3+^ (Figure 3B). The Ti^3+^/Ti^4+^ signal intensity ratio increases from 0.196 in pristine HTO to 0.212 in HTO‐PA and further to 0.355 in HTO‐HDA. Pillaring of HTO with PA or HDA therefore results in the reduction of the average Ti oxidation state, with more reduction in the case of HTO‐HDA. This could be explained by the overall higher number of amine groups in HTO‐HDA compared to HTO‐PA (ca. 2 versus 1.5 amine groups per H_2_Ti_4_O_9_, respectively). The N 1s core‐level spectra **(**Figure 3C) reveal the coexistence of NH_3_^+^ and NH_2_ species in the pillared materials, suggesting that the organic pillar exists in the structure in both its free and ionic (alkylammonium ion) forms.^[39]^ The ratio of NH_3_^+^ to NH_2_ was estimated to be ca. 4 to 1 in HTO‐PA, and 2.5 to 1 in HTO‐HDA, respectively. The higher NH_3_^+^/NH_2_ peak intensity ratio in HTO‐PA suggests a higher tendency for the organic pillars to exist in their ionic form compared to HTO‐HDA, where the alkyldiamine molecule might contain an ionized NH^3+^ on one end and a neutral NH_2_ on the other. Moreover, it is unlikely that covalent Ti−N bonds are forming due to the absence of a Ti−N peak at 397.2 eV.^[40]^ These findings agree with previous works that detected mixed NH_2_ and NH_3_^+^ species in alkylamine‐pillared titanates^[27]^ and alkyldiamine‐pillared vanadia.^[33]^

### 2.2. Electrochemical Characterization

With HTO, HTO‐PA and HTO‐HDA samples exhibiting highly comparable morphology and intralayer crystal structure, the influence of their interlayer properties on the lithium intercalation reaction can be analyzed. Specifically, the comparison of pristine versus pillared HTO allows to study the influence of interlayer spacing on the ion intercalation mechanism. For pillared HTOs, nanoconfined propylamine (PA) or hexanediamine (HDA) pillars lead to separated or cross‐linked titanate layers, respectively. With both HTO‐PA and HTO‐HDA exhibiting the same interlayer spacing, insights into the impact of electrochemo‐mechanics on the ion intercalation reaction can be gained.

**Figure 4:**
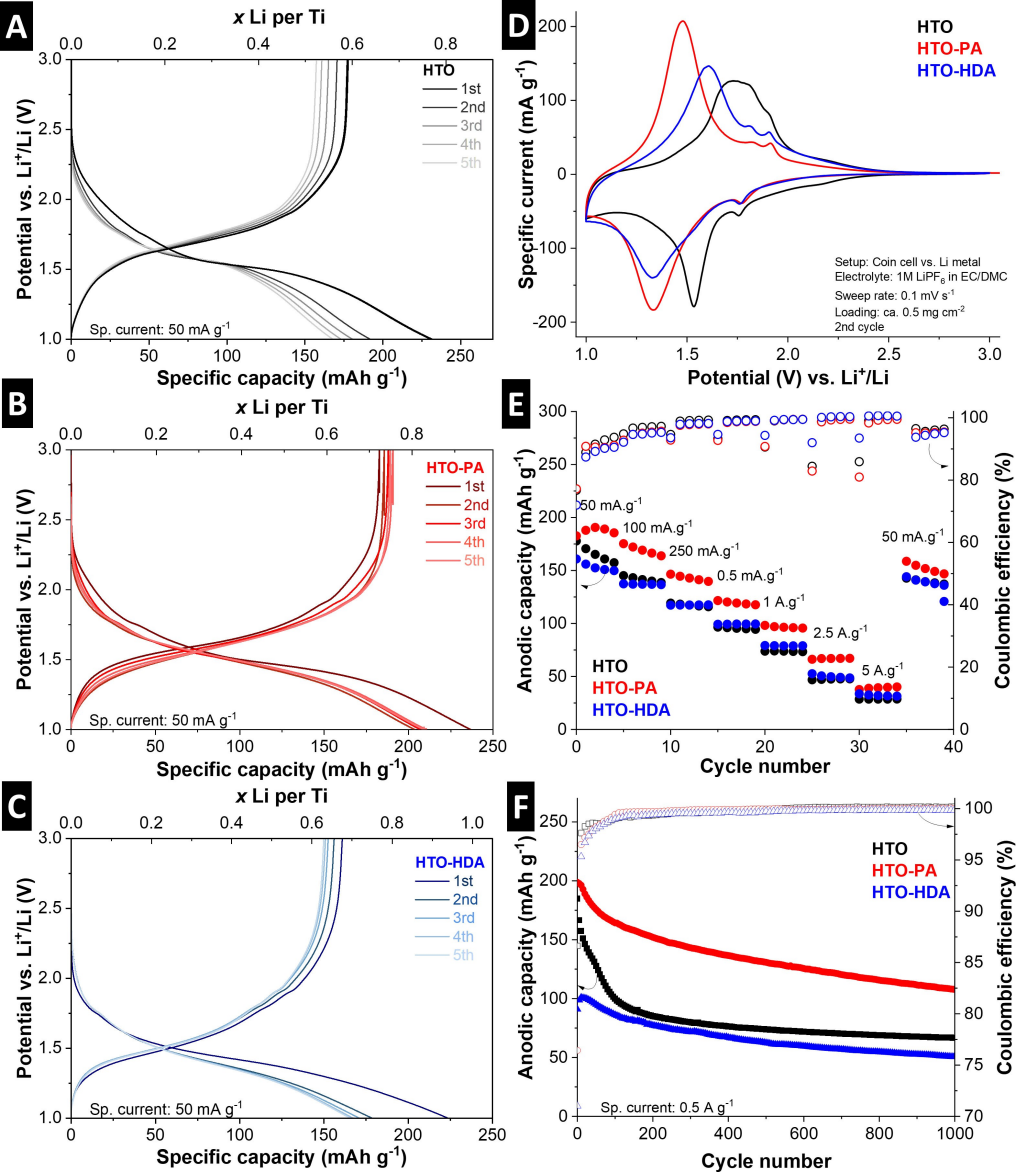
Electrochemical performance of HTO and pillared HTO in the Li‐ion system. (A−C) Galvanostatic charge and discharge plots of HTO, HTO‐PA, and HTO‐HDA showing the first 5 cycles at specific current rate 50 mA g^−1^. (D) CVs of HTO, HTO‐PA, and HTO‐HDA showing the 2^nd^ cycle, cycled at sweep rate 0.1 mV s^−1^. (E) Rate handling of HTO, HTO‐PA, and HTO‐HDA, showing anodic capacity at various values of specific current and the corresponding coulombic efficiency of each cycle. (F) Cycling performance of HTO, HTO‐PA, and HTO‐HDA at specific current rate 0.5 A g^−1^ and the corresponding coulombic efficiency. All measurements carried out in coin cells versus lithium metal with LP30 electrolyte at a constant temperature of 20 °C.

Galvanostatic charge/discharge (GCD) profiles of the first five cycles of HTO, HTO‐PA and HTO‐HDA at 50 mA g^−1^ in standard organic electrolyte (LP30) are shown in Figure 4A–C. All three samples exhibit a pronounced plateau for the lithium intercalation reaction. The reduction plateau is located at ca. 1.7 – 1.5 V for HTO and at ca. 1.5 – 1.3 V for HTO‐PA and HTO‐HDA, demonstrating a shift to a lower lithiation potential for pillared HTOs. All three samples exhibit comparable initial coulombic efficiencies (72–77 %) with slight performance degradation over the first five cycles (least pronounced for HTO‐PA). This indicates that organic pillars do not contribute to a decrease in initial efficiency or stability compared to pristine HTO.

Cyclic voltammograms at a sweep rate of 0.1 mV s^−1^ (Figure 4D) give additional insights into the (de)lithiation process. The shift to a lower lithiation (reduction) potential for PA‐ and HDA‐pillared materials is clearly visible. The position of the delithiation (oxidation) peak for the three materials is different, showing a variation of the overpotential of the (de)lithiation reaction. HTO‐PA shows the lowest overpotential (ca. 150 mV) compared to HTO‐HDA (ca. 280 mV) and HTO (ca. 220 mV), indicating the highest electrochemical reversibility for HTO‐PA. Additionally, there is an identical very low intensity set of redox peaks visible around 1.75–1.8 V for all samples. These can be attributed to (de)lithiation of anatase TiO_2_ impurity,^[41]^ indicative of minor secondary phases across all three titanate samples potentially resulting from trace amounts of TiO_2_ precursor from the solid‐state synthesis of K_2_Ti_4_O_9_. Since it is equally present in each sample, its influence on the overall comparability between the samples is negligible.

Quantitative analysis of the lithium intercalation capacity and kinetics is carried out by GCD at varying rates between 50–5,000 mA g^−1^ (Figure 4E). The specific delithiation capacities at low rates (fifth cycle) of HTO, HTO‐PA and HTO‐HDA are 157, 186 and 150 mAh g^−1^, respectively. There is a varying mass contribution of the different nanoconfined interlayer molecules in the three samples, which are not participating in the reversible charge storage process, namely 1.0 H_2_O, 1.5 PA, or 1.0 HDA. Normalizing the maximum reversible lithium storage capacities of the materials per tetratitanate can then be calculated as ca. 2.10 Li^+^ for HTO, 2.95 Li^+^ for HTO‐PA, and 2.52 Li^+^ for HTO‐HDA. This demonstrates that the maximum reversible ion storage capacity per transition metal is increased in interlayer‐expanded, organically pillared HTO materials. At increasing GCD rates, the performance of HTO‐PA remains superior compared to HTO and HTO‐HDA. This indicates superior charge storage kinetics when using the layer separating/non‐cross‐linking PA pillars compared to cross‐linking HDA pillars. However, the capacity retention of cross‐linked HTO‐HDA at high rates is still slightly higher compared to pristine HTO, indicating the general favorability of increased interlayer spacing for charge storage kinetics. This is also in line with previous reports of our group on alkyldiamine‐pillared vanadium oxide host materials.^[33]^

The cycling stability of HTO‐based materials is tested by GCD at an intermediate rate of 500 mA g^−1^ (Figure 4F). The performance degradation is most severe for pristine HTO, with a retention of 36 % of its initial capacity, while HTO‐PA and HTO‐HDA both retain about 55 % of their initial capacity after 1,000 cycles. The strong performance degradation for HTOs in organic lithium ion‐based systems has been observed before and attributed to hydrogen gas evolution at strongly reductive potentials.^[13]^ This adverse effect on the cycling stability appears to be weakened in HTO‐PA and HTO‐HDA. We hypothesize that this is due to a stabilization of confined structural protons due to NH^3+^ formation after introduction of amine groups. TEM images were recorded after cycling the show no visible signs of exfoliation or structural degradation (Figure S4). However, the (electrochemical) stability of confined interlayer species like protons, water, and organic pillars is still an underexplored topic and must be comprehensively analyzed to improve the cycling stability and Coulombic efficiency of pillared host materials.

Overall, the results demonstrate that the increased interlayer spacing of organically pillared HTOs results in an increased Li^+^ storage capacity per Ti compared to pristine HTO. Moreover, the layer separating PA pillars result in superior charge storage kinetics with increased electrochemical reversibility compared to cross‐linking HDA pillars.

**Figure 5:**
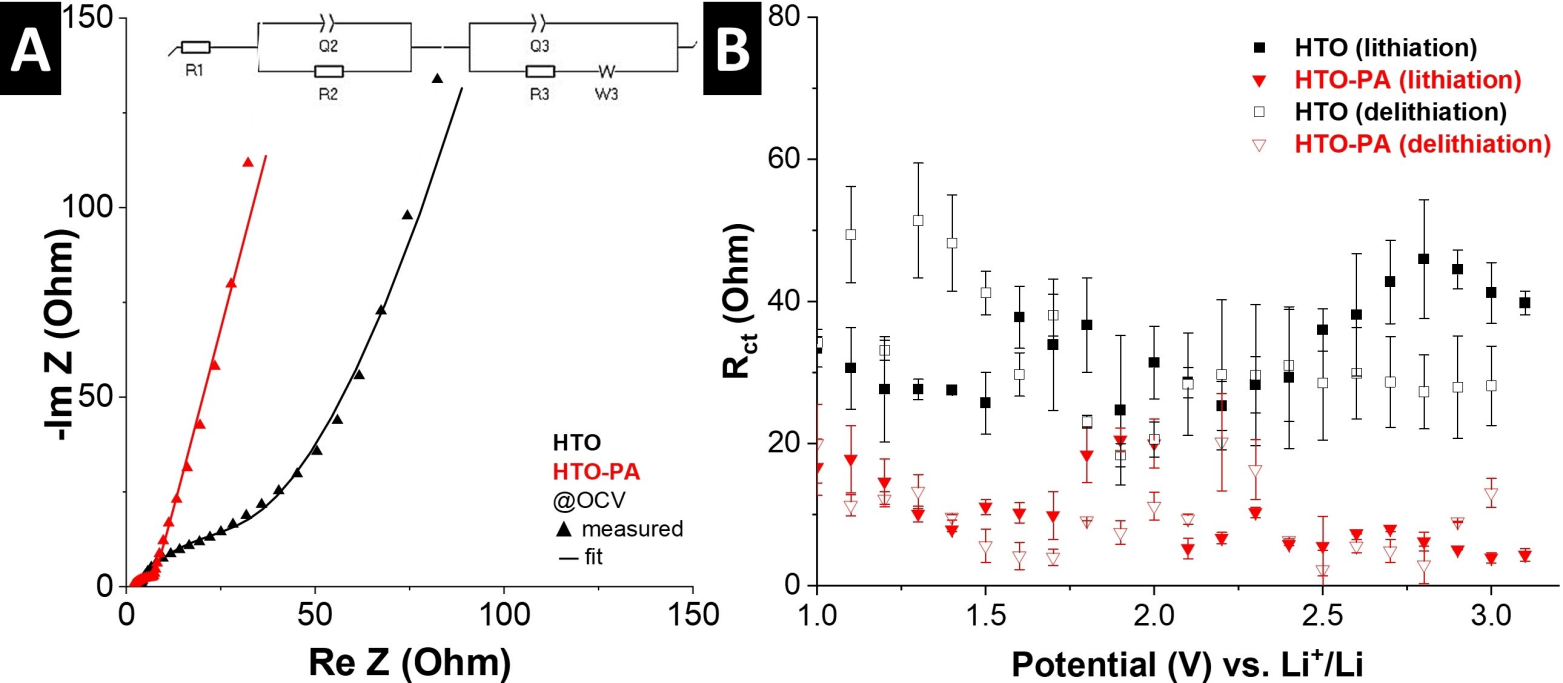
(A) Electrochemical impedance spectroscopy (EIS) Nyquist plots for HTO and HTO‐PA at OCV, measured in three‐electrode cells, fitted using the equivalent circuit shown in the inset. (B) R_ct_ values at different stages of lithiation and delithiation for HTO and HTO‐PA. Error bars are based on two measurements.

Favorable kinetics for ion intercalation in HTO host materials has been ascribed to ion‐solvent cointercalation phenomena before, when specially formulated electrolytes were used.^[17]^ We hypothesize that a nanoconfinement‐induced cointercalation mechanism is the origin of superior electrochemical kinetics of HTO‐PA compared to HTO, which is known to exhibit solid‐solution intercalation of desolvated Li^+^.^[13]^ Cointercalation reactions exhibit a reduced charge transfer resistance compared to the corresponding desolvated ion intercalation process, because of the circumvention of the desolvation step.[^42^, ^43^, ^44^, ^45^] Hence, we can probe this behavior using electrochemical impedance spectroscopy (EIS) at various states of charge (“staircase potentio electrochemical impedance spectroscopy”) in a three‐electrode cell setup. The Nyquist plots of HTO and HTO‐PA at open circuit voltage (OCV, ca. 3.1 V vs. Li^+^/Li) are shown in Figure 5A. An equivalent circuit consisting of the elements described in Figure S5 is used to model the impedance response of the intercalation process. Plotting the extracted charge transfer resistances clearly shows strongly reduced values for the charge storage process in HTO‐PA compared to HTO across the entire potential range for intercalation and deintercalation (Figure 5B). The results clearly support the assignment of the cointercalation mechanism in HTO‐PA electrodes.

**Figure 6:**
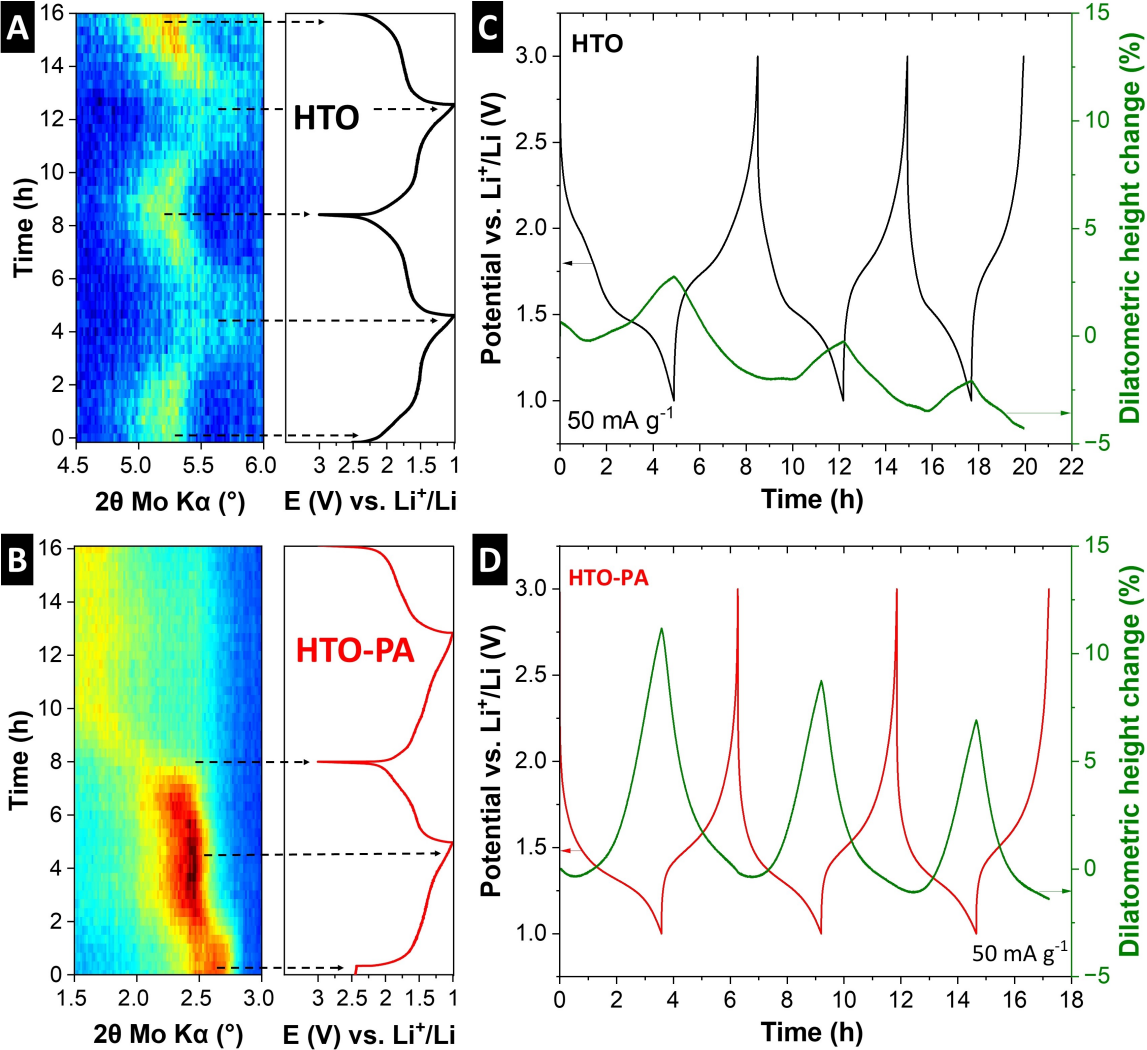
Operando X‐ray diffraction for (A) HTO and (B) HTO‐PA, highlighting changes to the (200) plane peak over 2 consecutive cycles of galvanostatic charge and discharge at a current rate of 50 mA g^‐1^. Operando electrochemical dilatometry measurement of (C) HTO, (D) HTO‐PA over 3 consecutive cycles at a current rate of 50 mA g^−1^.

To further support the hypothesis of a nanoconfinement‐induced cointercalation mechanism in HTO‐PA, the lithiation‐induced structural changes occurring in the different host materials are analyzed during electrochemical cycling using *operando* XRD. The electrode materials are cycled by GCD at 50 mA g^−1^ in a modified coin cell setup with Kapton‐sealed windows, while X‐ray diffractograms are continuously recorded in Debye‐Scherrer geometry (transmission) with a focus on the signal representative of the interlayer spacing, i. e., the (200) set of planes. For pristine HTO, a continuous shift towards smaller interlayer spacing is observed during electrochemical lithiation (Figure 6A), indicating a contraction of titanate layers upon insertion of a positive charge in the interlayer space. In the fully lithiated state, the HTO interlayer spacing contracted from ca. 0.8 to 0.74 nm. The interlayer spacing reversibly expands to its initial value after full electrochemical delithiation, suggesting a solid‐solution intercalation mechanism of Li^+^. The behavior is fully reversible and is in line with previous observations of interlayer contraction of titanates upon both proton or lithium insertion.[^13^, ^14^]

For HTO‐PA, the operando XRD profiles show a different behavior (Figure 6B). For interlayer‐expanded HTO‐PA, upon lithiation, the interlayer spacing further increases from 1.54 nm to ca. 1.68 nm. Upon delithiation, a further expansion to ca. 1.73 nm is observed. Similar observations are made for HTO‐HDA (Figure S6). Furthermore, in the second lithiation cycle, the signal corresponding to the (200) plane loses intensity for HTO‐PA, which indicates a loss of long‐range crystalline order. The observations support the insertion of a large intercalant in HTO‐PA (e. g., a partially solvated Li^+^), which apparently leads to stronger structural changes of the host lattice compared to pristine HTO. The results are fully in line with the transition from solid‐solution intercalation of desolvated Li^+^ in HTO to ion‐solvent cointercalation in HTO‐PA and HTO‐HDA. The intercalation of large ion‐solvent complexes qualitatively explains the observed further expansion of the interlayer spacing. Furthermore, this electrochemically‐induced expansion is likely facilitated by the non‐cross‐linked structure of HTO‐PA, explaining its superior kinetics compared to HTO‐HDA.

Given the difficulty of analyzing the structural changes of electrode materials with reduced crystalline long‐range order during electrochemical cycling with XRD, we employ complementary electrochemical dilatometry (ECD) analysis on HTO and HTO‐PA electrodes. The technique assesses the macroscopic height change of electrodes upon electrochemical (de)lithiation.^[46]^ This allows us to verify the hypothesis of a nanoconfinement‐induced cointercalation mechanism in HTO‐PA. Electrochemical (de)lithiation of HTO and HTO‐PA electrodes over five consecutive cycles at a rate of 50 mA g^−1^ and the associated dilatometric responses are shown in Figure 6C–D. The significant dilatometric height change in HTO‐PA is in line with previously identified cointercalation phenomena by ECD, such as the sodium‐diglyme system in graphite, though smaller in absolute magnitude.^[47]^ This can be explained by the already pre‐expanded structure of pillared HTO‐PA that can more readily accommodate (partially) solvated ions. It underlines that nanoconfinement‐design of host materials can address the issue of excessive volumetric change of cointercalation reactions that was identified, for example, in the graphite case.^[48]^

**Figure 7:**
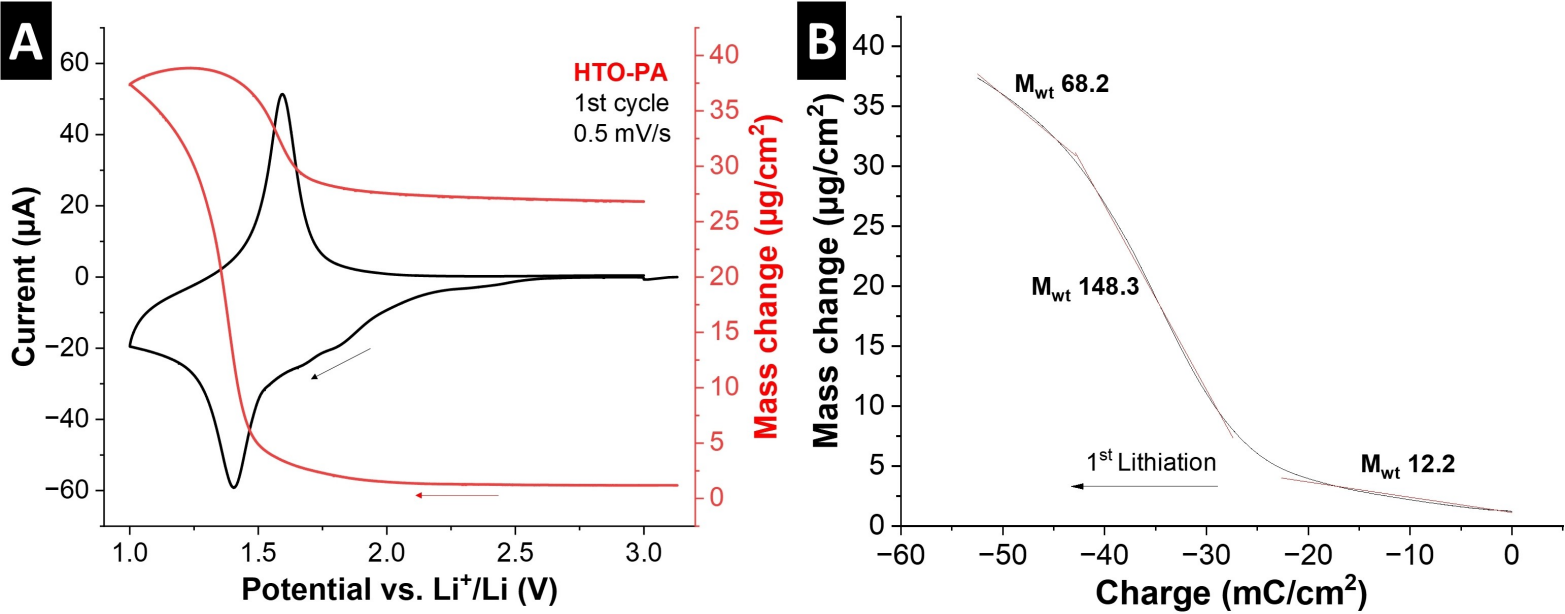
EQCM measurement of HTO‐PA. (A) Cyclic voltammogram of the first cycle of HTO‐PA in LP30 electrolyte at a sweep rate of 0.5  mV s^−1^, including the mass change derived from the change in resonance frequency. (B) Cumulative charge plotted versus mass change in the first cathodic cycle, with fitting of three linear regimes indicating different molar mass of the intercalant.

With strong indications of cointercalation phenomena in HTO‐PA given from the perspective of the host material response, we now employ electrochemical quartz crystal microbalance (EQCM) measurements to directly probe the nature of the (co)intercalating species in HTO‐PA. The method makes use of the linear relation between the change of resonance frequency of a gold‐coated quartz sensor and its mass according to the Sauerbrey equation.^[49]^ The linearity between frequency and mass change is only valid for ideally rigid coatings on the sensor, which is why we employ a thin film coating of active material and binder by drop‐casting. The dissipation of the crystal at different overtones was monitored in air, prior to cycling, to ensure the rigidity of the film. To verify the rigidity of the coating, values of changes in dissipation Δ*D* should be minimal and the frequency shift Δ*f* should be independent of overtone n (Figure S7).^[50]^ We verify the rigidity of coatings in air (Figure S7A) as well as during cycling in the electrochemical cell and in contact with organic electrolyte (Figure S7B), where the condition of ΔD/Δf <4*10^−7^ Hz^−1^ is met indicating validity of the Sauerbrey equation throughout electrochemical operation.[^51^, ^52^] This now allows us to investigate the mass change of the HTO‐PA electrode during electrochemical intercalation via cyclic voltammetry at 0.5 mV/s (Figure 7A). Plotting of the electrode mass change against the stored charge then allows for (semi−)quantitative analysis of the intercalant. Three distinct lithiation regimes (1‐3) are detected (Figure 7B). Initial lithiation occurs in the desolvated state (M_w,1_=12 g/mol), coinciding with the initial shrinking detected by ECD. Subsequently, a drastic increase in the intercalant mass is detected (M_w,2_=148 g/mol), indicating Li^+^ intercalation together with ca. 1.5 solvent molecules. Finally, the mass is decreased when the electrode is almost fully reduced, i. e., the interlayer space is almost completely filled (M_w,3_=68 g/mol). The similar mass of both solvents (EC and DMC) prevents us from directly identifying the involved solvent type. During delithiation, the reverse trend is observed. However, a significant hysteresis of the HTO‐PA mass change of 25.6 μg cm^−2^ (corresponds to 70.8 % of the initial mass gain) indicates that either some solvent remains confined in the interlayer space after Li^+^ deintercalation, or the accumulation of solvent molecules at the interface of the electrode. Separating the mass changes arising from solvent accumulation at the interface and solvent cointercalation in the electrode is notoriously challenging by EQCM. Previous EQCM work on Li^+^ intercalation from EC/DMC electrolyte into TiS_2_ demonstrated that solvent accumulation could lead to average molar mass changes between 20–35 g/mol,^[53]^ which is significantly below the mass changes detected in our experiments.

A hysteresis with continued mass gain persists over the following cycles, but decreases to 50.6 % and 40.7 % in the second and third cycles, respectively (Figure S8). These observations also suggest that no PA pillar dissolution (which would result in mass loss) takes place over the first few cycles.

Overall, the semi‐quantitative EQCM investigation demonstrates a cointercalation mechanism of partially solvated Li^+^ in HTO‐PA (4 solvent molecules assumed for full solvation of Li^+^ in EC/DMC mixture^[54]^). Overall, both EQCM and ECD results confirm the proposed ion‐solvent cointercalation mechanism in HTO‐PA, EQCM results suggest that solvent cointercalation is partially irreversible (due to mass hysteresis), but ECD results show a highly reversible swelling behavior without hysteresis. This highlights the necessity for a complete quantification of the chemical nature of the intercalant (precise solvation number and solvent type), requiring further spectroscopic analysis that must be carried out in future work.

## 3. Conclusions

A series of hydrogen titanates was synthesized that exhibits variations in (i) interlayer spacing from 0.88 to 1.58 nm and (ii) in the electrochemo‐mechanical properties of the interlayer space. The variations are based on the insertion of organic pillar molecules into HTO to expand the nanoconfined interlayer space, and by comparing non‐cross‐linking monoamine pillars (HTO‐PA) with cross‐linking diamine pillars (HTO‐HDA). Using spectroscopic, thermogravimetric, transmission electron microscopy and diffraction experiments in combination with density functional theory, we propose optimized atomistic structural configurations of the pillared HTO materials that show consistent interlayer spacings. It was shown that linear alkyl (di)amines act effectively as pillars, because their amine group(s) form ammonium cations that anchor to the HTO host lattice, and their alkyl backbone acts as a geometric spacer.

The electrochemical Li^+^ intercalation properties were evaluated in organic electrolyte as a function of the nanoconfinement properties of the HTO host materials. It was found that interlayer expansion by molecular pillars results in an increased maximum Li^+^ storage capacity from ca. 2.1 Li^+^ per tetratitanate in pristine HTO to ca. 2.95 Li^+^ per tetratitanate in HTO‐PA. The cathodic redox potential is shifted negatively by ca. 200 mV in pillared HTOs, indicating a modified electrode reaction. Moreover, the charge storage kinetics are most favorable when employing non‐cross‐linking PA pillars compared to cross‐linking HDA pillars or pristine HTO structure, yielding a reduced charge transfer resistance and increased reversibility. Employing electrochemical operando XRD, dilatometry and EQCM, we discovered that this is due to a nanoconfinement‐induced transition in intercalation mechanism from solid‐solution intercalation of desolvated Li^+^ in pristine HTO to cointercalation of partially solvated Li^+^ in pillared HTO.

This work demonstrates the potential of pillared host materials to achieve electrochemical cointercalation reactions. These materials exhibit favorable thermodynamic and kinetic properties, even with standard organic electrolytes like LP30. Future work should make use of the wide design space offered by organic molecules and focus on adjustments to the pillar chemistry, e. g., replacing the alkyl backbone with polyether or aromatic molecule backbones. This has the potential to further tune and optimize the functionality of pillared host materials, in particular regarding the interaction with electrochemically intercalating ions/ion‐solvent complexes.

## Conflict of Interests

The authors declare no conflicts of interest.

## Supporting information

As a service to our authors and readers, this journal provides supporting information supplied by the authors. Such materials are peer reviewed and may be re‐organized for online delivery, but are not copy‐edited or typeset. Technical support issues arising from supporting information (other than missing files) should be addressed to the authors.

Supporting Information

## Data Availability

All experimental and computational data is made available on the Zenodo repository, under DOI: 10.5281/zenodo.14258609 (https://doi.org/10.5281/zenodo.14258609).
