# Supplementary material for: Nanoconfinement‐Induced Electrochemical Ion‐Solvent Cointercalation in Pillared Titanate Host Materials
Source: Angew Chem Int Ed Engl. 2025 Mar 11;64(20):e202423593. doi: 10.1002/anie.202423593 (PMC12070453; doi:10.1002/anie.202423593)
Supplement: Supplementary file 1 — Supporting Information [file ANIE-64-e202423593-s001.pdf]

Angewandte Chemie
Eine Zeitschrift der Gesellschaft Deutscher Chemiker

Supporting Information

Nanoconfinement-Induced Electrochemical Ion-Solvent Cointercalation in Pillared Titanate Host Materials

*M. Elmanzalawy, H. Song, M. Tobis, R. Leiter, J. Choi, H. Moon, W.-Y. Tsai, D.-e. Jiang, S. Fleischmann**

Supporting Information

Nanoconfinement-induced Electrochemical Ion-Solvent Cointercalation in Pillared Titanate Host Materials

Mennatalla Elmanzalawy,[1,2] Haohong Song,[3] Maciej Tobis,[1,2] Robert Leiter,[1,2]

Jaehoon Choi,[1,2] Hyein Moon,[1,2] Wan-Yu Tsai,[4,5] De-en Jiang,[6] Simon Fleischmann[1,2,*]

[1] Helmholtz Institute Ulm (HIU), 89081 Ulm, Germany

[2] Karlsruhe Institute of Technology (KIT), 76021 Karlsruhe, Germany

[3] Interdisciplinary Materials Science, Vanderbilt University, Nashville, Tennessee, 37235, United States

[4] Univ. Lille, CNRS, Univ. Polytechnique Hauts-de-France, UMR 8520 - IEMN, F-59000 Lille, France

[5] Réseau sur le Stockage Electrochimique de l'Energie (RS2E), CNRS FR 3459, 33 rue Saint Leu, Amiens, Cedex 80039, France

[6] Department of Chemical and Biomolecular Engineering, Vanderbilt University, Nashville, Tennessee, 37235, United States

[*] Corresponding author's email: simon.fleischmann@kit.edu

1. Experimental methods

Material synthesis

Hydrogen tetratitanates ($H_2Ti_4O_9{\cdot}H_2O$, hereon referred to as HTO) was synthesized as per the process reported by Izawa et al. where first a potassium tetratitanate ($K_2Ti_4O_9$) precursor was obtained by heating a mixture of potassium carbonates (K_2CO_3, VWR Chemicals) and titanium (IV) oxide (TiO_2, Thermo Scientific) in a 1:3.5 molar ratio at 800 °C for 20 h in a muffle furnace (Nabertherm P330) and then another 20h after grinding with a mortar and pestle.[1] Then, to obtain HTO, 1 g of the obtained $K_2Ti_4O_9$ was stirred in 200 mL of 1 M hydrochloric acid (HCl, VWR Chemicals) for 3 days at 60 °C. The acid solution was changed daily to remove the exchanged K^+. The obtained product was filtered and dried at room temperature under air flow before subsequent characterization.

To obtain alkylamine-pillared HTO, the HTO powder was stirred in aqueous solutions of the alkylamine at room temperature for 3 days as per a modified protocol similar to Izawa's.[1] For HTO-PA, 200 mg of HTO powder was added to 6.66 ml of a 50 vol.% aqueous solution of propylamine (C_3H_9N, Thermo Scientific). For HTO-HDA, 200 mg of HTO were added to 6.66 ml of 60 vol.% aqueous solution of 1,6-Hexanediamine ($C_6H_{16}N_2$, VWR Chemicals). The obtained powder was filtered and washed with deionized water several times until the pH of the solution was neutral, then left to dry in air at room temperature.

Structural and morphological characterization

Powder X-ray diffraction (XRD) patterns of the samples were obtained using a Bruker D8 Advance equipped with a Cu K_α radiation source (λ = 0.15406 nm). The patterns were recorded in Bragg-Brentano mode in the 2θ range between 2° and 40° 2θ with 0.02° step size.

Scanning electron microscopy (SEM) imaging was performed using a ZEISS Crossbeam 340 electron microscope operated at 5 kV and working distance of 7.4 mm. Raman spectra were obtained using a Renishaw Invia Raman spectrometer equipped with an He-Ne laser with a wavelength of 633 nm using 5 mW laser power. Thermogravimetric analysis of the powder samples was performed using a NETZSCH TG 209 F1 Libra thermal analyzer under O_2/N_2 flow at a heating rate of 5 K min^{-1} in Al crucibles loaded with ca. 5 mg of material.

Inductively Coupled Plasma (ICP) analysis was performed using a Spectro ARCOS FHS12 inductively coupled plasma optical emission spectrometer (ICP-OES). The sample was dissolved in aqua regia (mixture of $HCl:HNO_3$ with molar ratio 3:1), and treated in a microwave twice to dissolve the solid powder particles.

X-ray photoelectron spectroscopy (XPS) of the synthesized powder samples was performed in a fixed analyzer transmission mode, using a monochromatic Al Kα (hν = 1.487 eV) X-ray and a Phoibos 150 XPS spectrometer (Surface Concept) with a micro-channel plate and Delay Line Detector (DLD). High resolution Ti 2p, C 1s, N 1s, and O 1s regions were acquired with an X-ray power source of 200 W (12 kV), pass energy of 30 eV, and 0.1 eV energy steps. The spectra analysis was carried out by CasaXPS software, using a nonlinear Shirley-type background.

Transmission Electron Microscopy (TEM) was performed using a ThermoFisher Talos F200i TEM at a low acceleration voltage of 80 kV. Samples were prepared for TEM analysis by collecting a small amount of powder on a holey carbon TEM grid. For TEM analysis of materials post cycling, electrodes were recovered from coin cells and washed in an Ar-filled glovebox with DMC solvent and dried for 5 minutes in the vacuum of the glovebox transfer chamber. Then, material was removed from the dried electrode sheets using a sharp blade, and the TEM grid was gently rubbed over the collected powder.

Density functional theory (DFT) computation

DFT calculations were performed using the Vienna ab initio Simulation Package (VASP)[2,3] with the Perdew–Burke–Ernzerhof (PBE)[4] form of the generalized-gradient-approximation exchange correlation functional. The projector augmented wave (PAW) potential was used to describe the electron–nuclei interaction.[5] An energy cutoff of 450 eV was used for the plane wave basis sets. Based on the ratios of HTO to HDA (1:1), HTO to PA (1:1.5), and the different symmetries in the distribution of PA, we constructed the structures as follows. The structures of HTO-HDA were simulated by a supercell containing 4 $H_2Ti_4O_9$ and 4 $NH_2C_6H_{12}NH_2$ formula units; the HTO-PA structures were simulated by a supercell containing 4 $H_2Ti_4O_9$ and 6 $C_3H_7NH_2$ formula units or 8 $H_2Ti_4O_9$ and 12 $C_3H_7NH_2$ formula units. All degrees of freedom were relaxed with a force convergence criterion of 0.05 eV $Å^{-1}$ and energy convergence criterion of 10^{-5} eV. A 1 × 2 × 2 Monkhorst–Pack k point grid was adopted for Brillouin zone integration.[6] DFT-D3 was used to account for the van der Waals interaction.[7]

Electrochemical characterization

The synthesized powder samples were used to make electrode slurries where the active material, conductive carbon (C65, C.Energy), and polyvinylidene fluoride (PVdF, Solvay) binder (2 wt.% PVDF dispersed in N-Methyl-2-pyrrolidone, NMP, Thermo Scientific) were mixed in an 8:1:1 mass ratio. The slurries were then cast on carbon-coated aluminium current collector (20 µm, battery grade, Welcos) using a doctor blade, and the wet thickness was set to 60 µm. The cast electrodes were then left to dry overnight in circulating air (fume hood) at room temperature, and then further in vacuum at room temperature overnight.

Electrochemical measurements were performed in 2032-type coin cells versus 12 mm diameter metallic Li (500 µm thickness, Honjo) disc electrodes, LP30 (1M $LiPF_6$ in 1:1 vol.% EC:DMC, Solvionic) was used as electrolyte, and a 19 mm diameter Whatman glass microfiber (grade GF/A) was used as a separator. For electrochemical impedance measurements, three-electrode cells (stainless-steel Swagelok-type "T-cells") were used with a 12 mm diameter Li metal disk counter electrode and a 5 mm diameter Li metal disk as quasi-reference electrode and a 13 mm diameter Whatman glass microfiber disk separator.

To make electrode slurries for *in situ* X-ray diffraction measurements, the active material powder, conductive C65 carbon, and PVDF were mixed in 7:2:1 mass ratio. The slurry was drop-cast onto a 12 mm diameter Ti mesh, achieving a mass loading of ca. 5 mg cm^{-2}, and left to dry under air circulation, then later under vacuum at room temperature. *In situ* X-ray diffraction patterns were recorded during galvanostatic cycling of the cast electrodes against a Li metal electrode in LP30 electrolyte with a current of 50 mA g^{-1} using a Bio-Logic SP-300 potentiostat, in a coin cell setup with 6 mm diameter Kapton windows on both sides of the coin cell. The diffractograms were recorded using a STOE STADI-p diffractometer in transmission geometry with Mo K_α radiation (λ = 0.07093 nm), equipped with a DECTRIS MYTHEN 1 K strip detector.

Staircase potential electrochemical impedance spectroscopy (SPEIS) measurements in the Li-ion system were recorded using 3-electrode T-cells in the frequency range 200 kHz to 100 mHz, with a voltage amplitude of 10 mV. The potential was changed from OCV to 1 V vs. Li^+/Li in 100 mV increments, with a 2-hour holding step at each potential prior to recording an impedance spectrum.

Galvanostatic intermittent titration technique (GITT) measurements were conducted in coin cells after one galvanostatic cycle at 50 mA g^{-1} current rate. The measurements were performed by applying a 30-minute, 20 mA g^{-1} current pulse followed by a 2-hour rest. Internal resistance was calculated by dividing the overpotential (difference between the voltage at the end of the current pulse step and the voltage at the end of the relaxation step) by the applied current.

Electrochemical dilatometry (ECD) measurements were conducted using an ECD-4-nano dilatometer (EL-CELL). Free-standing electrodes were prepared by mixing the active material, conductive carbon (C-NERGY Super C65, Imerys), and polytetrafluoroethylene (PTFE, 60 wt.% aqueous solution, Sigma-Aldrich) in a ratio of 80 wt.%, 10 wt.%, and 10 wt.%, respectively. Once the mixture achieved a dough-like consistency, it was calendared to form a sheet with a uniform thickness of ca. 50 µm. The sheet was then dried under vacuum at 60 °C, and 8 mm diameter electrodes were subsequently cut. After assembly, the ECD cell was allowed to rest at open circuit potential (OCP) for 6 hours before the electrochemical measurements. All ECD measurements were carried out in a climate-controlled chamber at 22 °C.

Electrochemical quartz crystal microbalance (EQCM) experiments were carried out with an AWS X1 device with temperature control (AWSensors) in combination with Bio-Logic SP-300 potentiostat with the temperature control unit of the QCM set to 23 °C. Electrodes were prepared by drop casting 35 µl of a dispersion of HTO-PA onto a gold-coated (Au with Ti adhesion layer) 5 MHz quartz crystal (AWSensors). The dispersions consisted of 95 wt.% active material powder and 5 wt.% PVDF binder dispersed in NMP at 2 mg ml^{-1} concentration. This resulted in active material loadings of ca. 70 µg cm^{-2}. Dispersions were sonicated for 2 hours in an ice-cooled sonication bath prior to drop casting. Hermetically sealed EQCM cells were assembled in a glovebox, with Li metal strip as CE/RE and 1M $LiClO_4$ in EC:DMC as electrolyte. The hexafluorophosphate salt from typical LP30 electrolyte must be avoided in EQCM experiments due to side reactions with the gold coating of the sensor.

2. Supplementary data

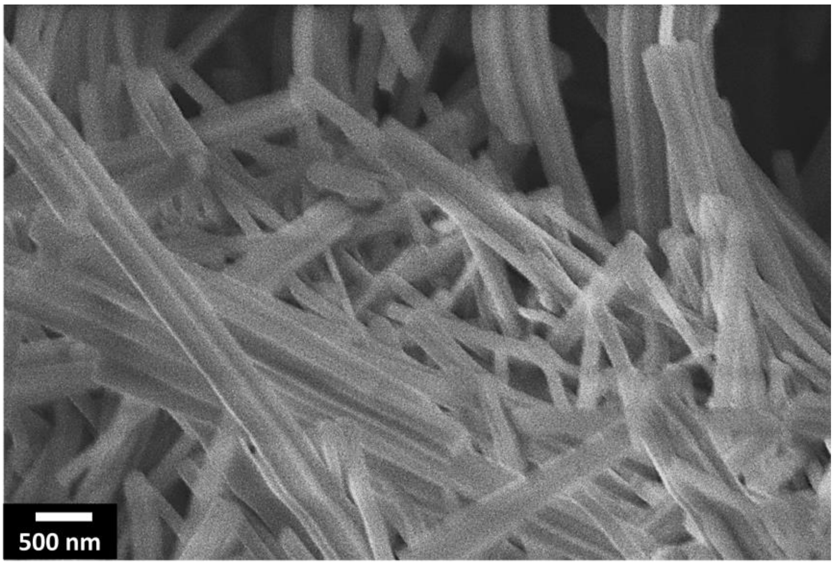

Fig. S1: Scanning electron micrograph of as-synthesized $K_2Ti_4O_9$, obtained from solid-state synthesis of TiO_2 with K_2CO_3. The material serves as the precursor for $H_2Ti_4O_9$.

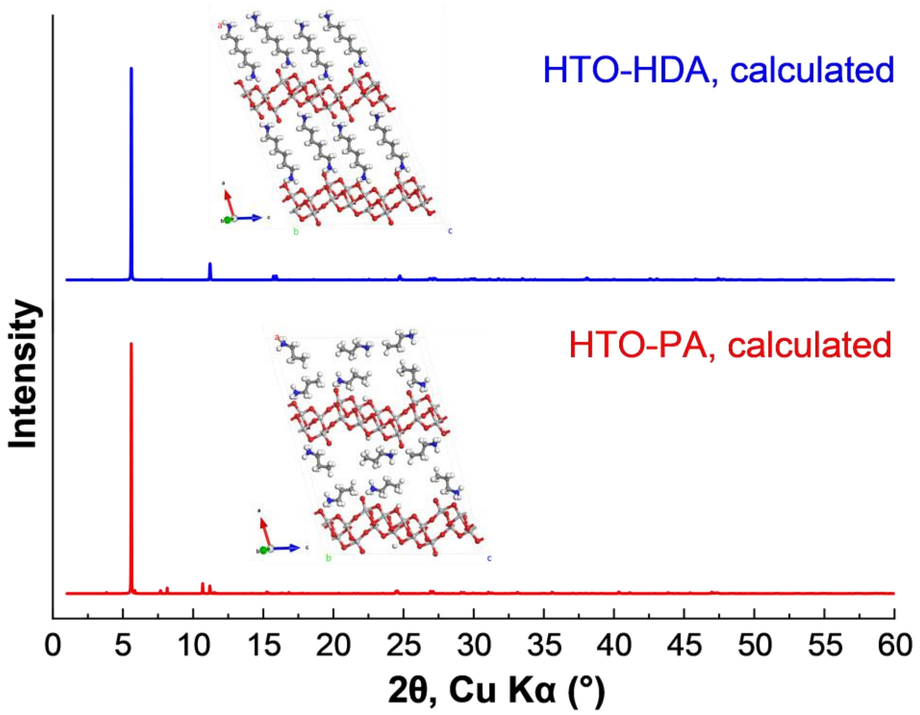

Fig. S2: Calculated XRD patterns of HTO-PA and HTO-HDA, based on DFT-optimized structures (their coordinates are provided at the end of this file in the VASP POSCAR format).

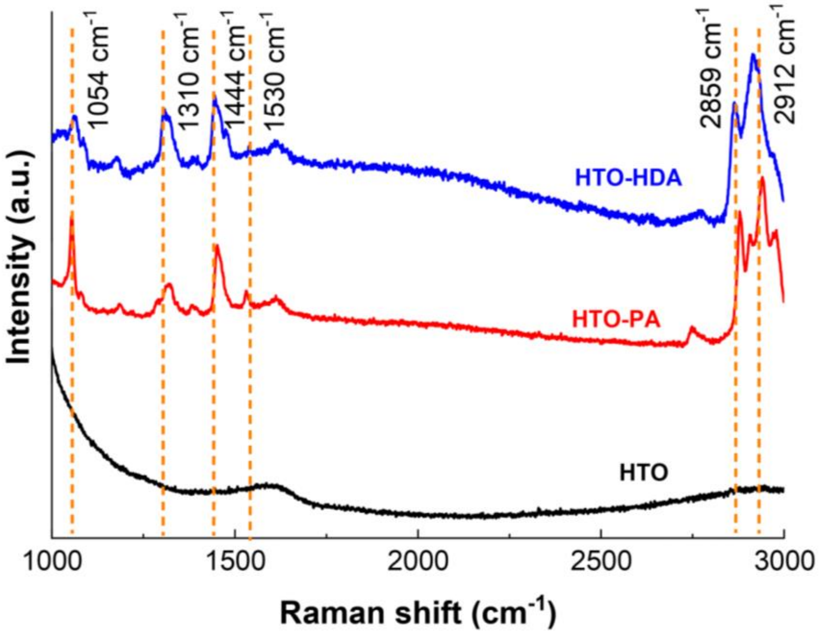

Fig. S3: Raman spectra in the range 1000 to 3000 cm^{-1}, showing the peaks characteristic of the organic pillar molecules.

In pillared HTO-PA and HTO-HDA, spectral features relating to the organic components are detected, which are absent in pristine HTO. Particularly, the peaks at 1054, 1310, 1444 cm^{-1} are characteristic of C-N stretching, C–C–H, and CH_2 vibrations, respectively.[8,9] Low intensity peaks at 1530 cm^{-1} are assigned to ammonium bending mode,[10] which is consistent with previous reports stating that *n*-alkylamines are present in the interlayer of titanate compounds in their cationic forms.[8] The high wavenumber bands at 2859 and 2912 cm^{-1} can be assigned to CH_2 symmetric and CH_2 asymmetric stretching modes, respectively.[11]

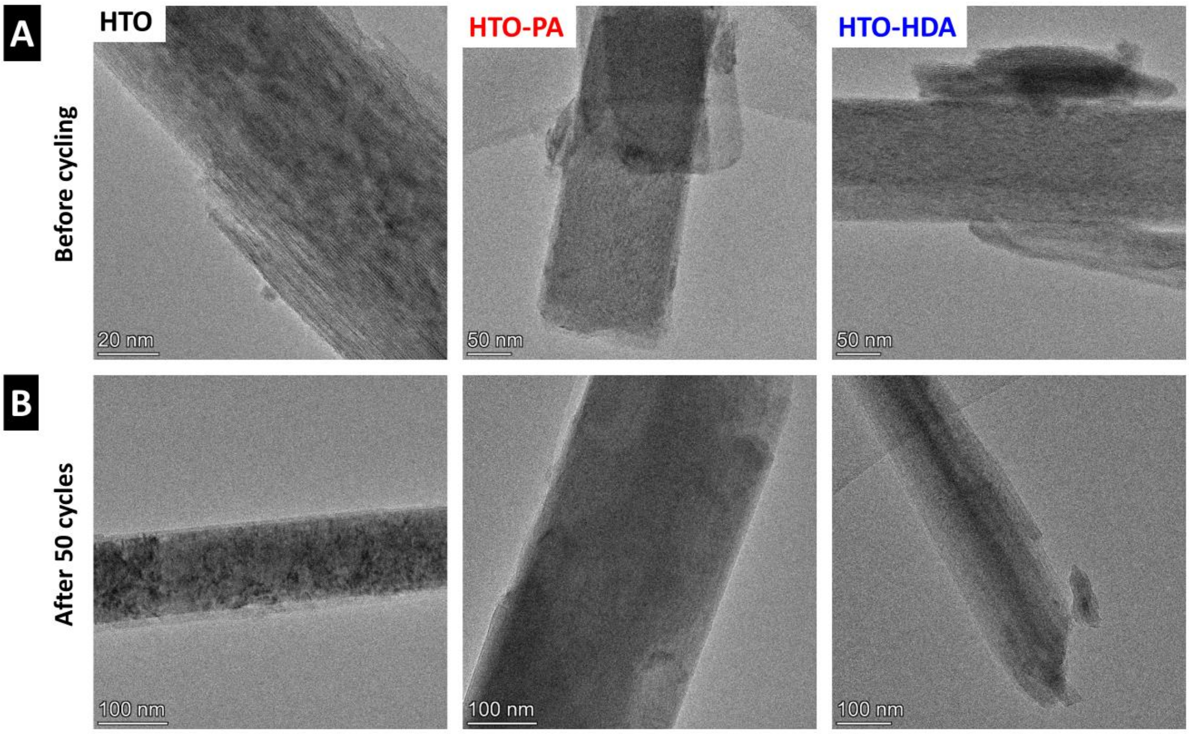

Fig. S4: TEM images of HTO, HTO-PA, and HTO-HDA electrodes (A) before cycling, and (B) after 50 cycles. The rod-like morphology is maintained after cycling and no signs of exfoliation or structural degradation are visible.

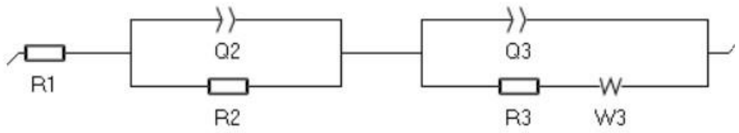

Fig. S5: Equivalent circuit used in the fitting of impedance spectroscopy data shown in this work. R1: Ohmic resistance of the cell, compromising electrolyte, separator, and electrodes. R2, Q2: resistance and capacitance of the interfacial layer, respectively. R3: charge transfer resistance, Q3: double-layer capacitance, and W3: Warburg diffusion element. Constant-phase elements (Q) are used instead of capacitors (C) to represent non-ideal double layer behavior.[12]

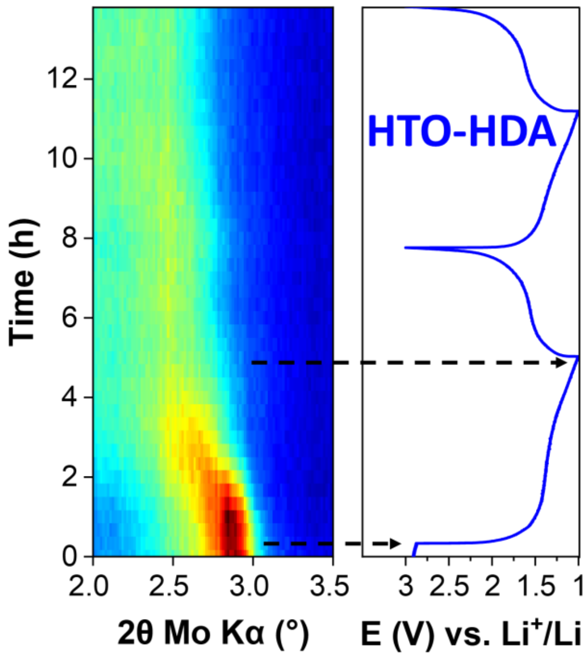

Fig. S6: Operando X-ray diffraction for HTO-HDA, highlighting changes to the (200) plane peak over 2 consecutive cycles of galvanostatic charge and discharge at a current rate of 50 mA g^{-1}.

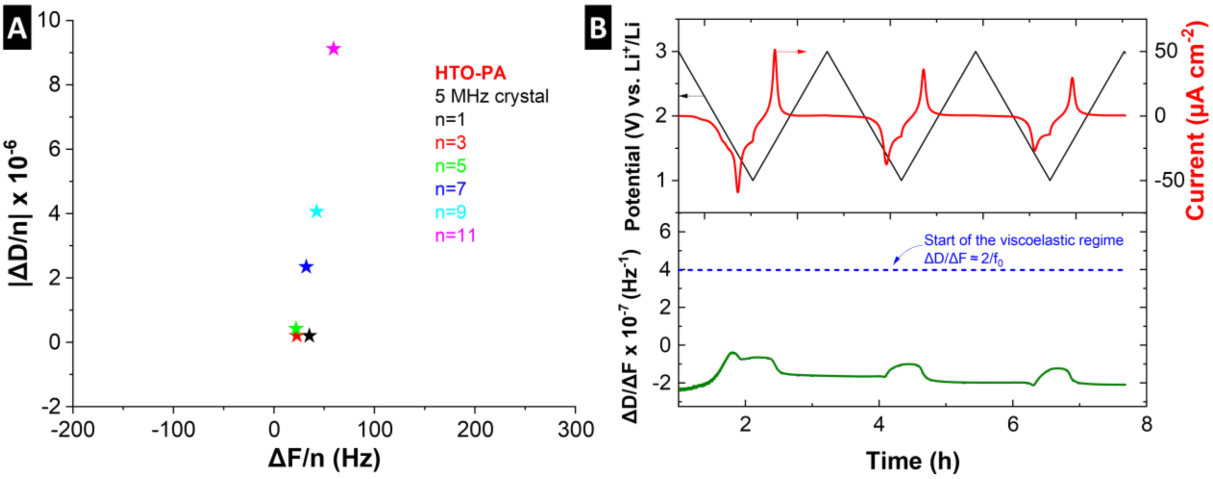

Fig. S7: (A) Dissipation (ΔD) vs. frequency change (Δf) plots verifying the rigidity of HTO-PA coatings on the quartz crystal. (B) Potential-current response of HTO-PA during the cyclic voltammetry experiment at 0.5 mV s^{-1} with the change of dissipation divided by the change of frequency during cycling. The start of the viscoelastic regime is indicated by the dashed blue line and defined as $2/f_0 = 4x10^{-7}$ Hz^{-1}, with f_0 being the fundamental frequency of the quartz crystal sensor (5 MHz).

For a rigid coating, where the Sauerbrey equation can be applied, the frequency shift of the electrode, in air, should be independent of n, and the dissipation factor changes D/n should be relatively small across all overtones. The overtone number, n, refers to the multiple of the fundamental frequency the crystal is vibrating at. For example, in this work, the fundamental frequency ($n = 1$) of the crystal is 5 MHz, hence the 3rd overtone ($n = 3$) would be 15 MHz, and so on.[13]

During cycling, as shown in Fig. S7B, the condition $\Delta D/\Delta f < 4*10^{-7}$ Hz^{-1} is met, meaning that we stay in the rigid regime and the Sauerbrey equation is applicable.[14,15]

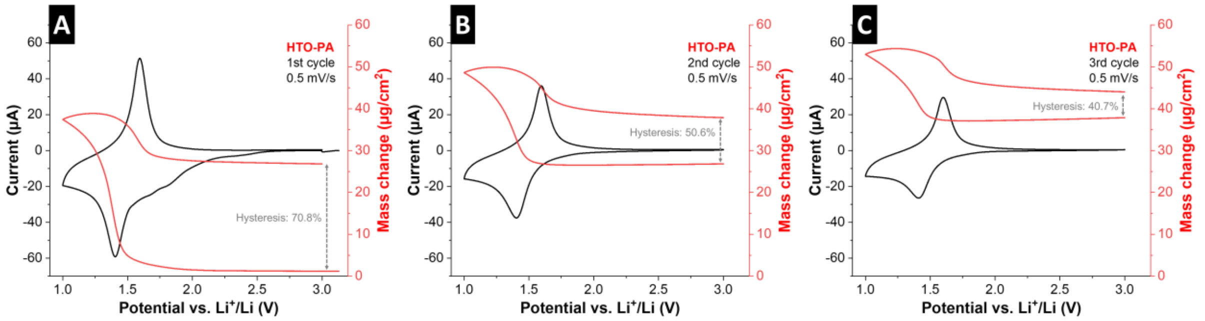

Fig. S8: EQCM measurement of HTO-PA of the (A) first, (B) second, and (C) third cycle in LP30 electrolyte at a sweep rate of 0.5 mV/s, including the mass change derived from the change in resonance frequency. The hysteresis is calculated as the fraction of mass retention (after the cycle) and mass gain (in %).

Table S1: ICP analysis of elements present in as-synthesized HTO, showing only trace amounts of K, indicating successful proton exchange.

**Sample**	**Element**	**Mass (%)**
HTO	K	0.22
	Ti	52.1

DFT-optimized structures for HTO, HTO-PA, and HTO-HDA in the VASP POSCAR format

HTO

```
**********************************
HTO: H8Ti16O36
1.0
      16.5984115601        0.0000000000        0.0000000000
       0.0014781254        3.8185526848        0.0000000000
      -3.9269981202        0.0033815039       11.3710129290
   O   Ti    H
  36   16    8
Direct
     0.005309000         0.000272000         0.370220989
     0.053950999         0.000645000         0.164320007
     0.154877007         0.001018000         0.734793007
     0.180111006         0.000440000         0.516258001
     0.220194995         0.000344000         0.294975013
     0.267944008         0.000376000         0.078732997
     0.364578009         0.000368000         0.653330982
     0.680491984         0.000206000         0.133120999
     0.896436989         0.999823987         0.052850001
     0.505268991         0.500063002         0.369731009
     0.554036021         0.500352979         0.164010003
     0.654893994         0.500737011         0.734549999
     0.680060029         0.500244021         0.516029000
     0.720270991         0.500048995         0.294768006
     0.768006980         0.500051022         0.078477003
     0.864571989         0.500321984         0.653025985
     0.180454001         0.500527024         0.133333996
     0.396400005         0.500088990         0.053146001
     0.993816972         0.000679000         0.629579008
     0.945186973         0.000004000         0.835350990
     0.844341993         0.999553025         0.264871001
     0.819067001         0.000247000         0.483368009
     0.778918028         0.000386000         0.704617023
     0.731307030         0.000280000         0.920966029
     0.634586990         0.000147000         0.346316993
     0.318722010         0.000386000         0.866559029
     0.102719001         0.000797000         0.946703017
     0.493808001         0.500262976         0.629657984
     0.445194989         0.500150979         0.835552990
     0.344289988         0.499909997         0.265092999
     0.319050997         0.500380993         0.483653009
     0.278966993         0.500501990         0.704905987
     0.231233001         0.500558972         0.921124995
     0.134575993         0.500424027         0.346547008
     0.818747997         0.500177979         0.866295993
     0.602833986         0.500478029         0.946587980
     0.106839001         0.000417000         0.353606999
     0.146246001         0.000583000         0.121166997
     0.246444002         0.000523000         0.663025975
     0.797104001         0.999922991         0.107675999
     0.606881022         0.500127971         0.353345007
     0.646317005         0.500245988         0.120975003
     0.746406019         0.500413001         0.662769020
     0.296965003         0.500285029         0.107831001
     0.892247021         0.000398000         0.646025002
     0.852940977         0.000106000         0.878485024
     0.752761006         0.000023000         0.336602002
     0.202156007         0.000671000         0.891972005
     0.392260998         0.500325024         0.646272004
     0.352955014         0.500325978         0.878817022
```

0.252692997	0.500311017	0.336917013
0.702148974	0.500379026	0.891776979
0.441118985	0.499783009	0.335763007
0.460992008	0.500136971	0.103188999
0.941173017	0.999705017	0.336124003
0.961018980	0.000064000	0.103169002
0.557922006	0.500585973	0.663612008
0.538218975	0.500383973	0.896489024
0.057962999	0.001170000	0.663716018
0.038183000	0.000514000	0.896552026

****

HTO-PA

****

HTO-PA: $(H_2Ti_4O_9)_8(C_3H_7NH_2)_{12}$

1.0

32.8832740784	0.0000000000	0.0000000000
-0.0265450469	3.8667568780	0.0000000000
-6.6328085625	-0.0438679252	23.0300620865

O Ti H C N

72 32 124 36 12

Direct

0.011349000	0.000090000	0.210406005
0.756869018	0.013032000	0.322409004
0.512151003	0.495373994	0.204784006
0.256217003	0.523211002	0.328435004
0.030796999	0.004253000	0.095821999
0.721327007	0.013128000	0.411547989
0.529780984	0.497963011	0.089346997
0.221527994	0.522442997	0.419028997
0.082762003	0.015121000	0.377855003
0.672967017	0.006383000	0.127354994
0.582846999	0.504133999	0.369955003
0.173849002	0.513221025	0.135518000
0.099496998	0.011829000	0.268094003
0.668551981	0.007881000	0.240468994
0.600458980	0.503214002	0.260363996
0.167977005	0.517036021	0.248200998
0.117232002	0.009860000	0.155467004
0.641762972	0.006948000	0.350959986
0.616394997	0.502939999	0.147297993
0.141745001	0.517931998	0.358615994
0.139402002	0.010658000	0.048734002
0.615483999	0.009954000	0.459867001
0.638068020	0.504274011	0.040755998
0.115975998	0.521238983	0.467702001
0.188881993	0.020056000	0.332780987
0.572741985	0.000504000	0.178072006
0.689222991	0.509962976	0.325462013
0.072788000	0.507296026	0.185041994
0.588690996	0.001082000	0.065699004
0.164032996	0.021864999	0.441967994
0.090034999	0.507786989	0.073895998
0.663975000	0.510210991	0.434601992
0.697651982	0.008241000	0.021195000
0.054037999	0.006212000	0.485410988
0.198882997	0.514254987	0.028907999
0.553691030	0.494001001	0.477477014
0.999467015	0.004512000	0.687631011
0.744153976	0.025985001	0.799952984
0.498757988	0.498338014	0.678326011
0.245078996	0.509459019	0.807461023

0.034619998	0.012044000	0.597544014
0.723991990	0.010498000	0.913183987
0.533990026	0.502113998	0.589308977
0.224756002	0.516182005	0.920670986
0.082011998	0.007448000	0.880993009
0.672554016	0.009538000	0.630762994
0.580884993	0.501555979	0.873157978
0.173168004	0.519087970	0.638410985
0.087886997	0.011054000	0.768153012
0.656042993	0.007754000	0.740781009
0.586853981	0.504339993	0.760482013
0.156798005	0.513958991	0.748360991
0.114389002	0.014573000	0.657815993
0.637663007	0.006677000	0.853438020
0.613699973	0.505620003	0.650026023
0.138594002	0.510406017	0.861109018
0.139895007	0.022577001	0.548560023
0.615180016	0.003155000	0.959896028
0.639805973	0.511388004	0.540965021
0.116305999	0.509173989	0.967857003
0.183044001	0.012587000	0.831453025
0.566578984	0.003227000	0.675756991
0.682111025	0.510342002	0.823903978
0.067138001	0.511407971	0.683664978
0.591311991	0.006115000	0.566231012
0.165726006	0.012126000	0.942861021
0.091805004	0.516525984	0.574240983
0.664633989	0.506944001	0.935068011
0.701569974	0.001760000	0.523397982
0.056910001	0.005181000	0.987950027
0.201887995	0.522157013	0.530947030
0.555459976	0.497940987	0.979532003
0.058263998	0.005589000	0.192967996
0.702480972	0.010472000	0.321110994
0.558528006	0.499375999	0.185837999
0.202079996	0.520363986	0.328561991
0.079901002	0.007140000	0.074153997
0.675328016	0.015610000	0.434399009
0.578123987	0.500374019	0.065938003
0.175580993	0.526669025	0.442032009
0.129010007	0.017107001	0.339881986
0.631268024	0.004165000	0.171541005
0.629404008	0.506538987	0.332347989
0.131400004	0.511335015	0.178918004
0.648513019	0.004776000	0.050781000
0.104906000	0.029090000	0.453696012
0.150041997	0.511227012	0.058867000
0.604605973	0.517874002	0.445807993
0.053606000	0.009299000	0.687702000
0.696910977	0.012997000	0.816455007
0.553189993	0.502089977	0.679776013
0.197768003	0.511937976	0.823840022
0.080546997	0.020056000	0.574443996
0.674965024	0.007888000	0.935091019
0.580039978	0.510109007	0.566601992
0.175772995	0.512718022	0.942595005
0.124408998	0.010012000	0.837454021
0.626492977	0.006694000	0.669066012
0.623440981	0.506224990	0.829883993
0.126980007	0.515345991	0.676721990
0.650541008	0.020761000	0.554906011
0.105636999	0.008444000	0.957642972
0.151014000	0.530745983	0.562650025

0.604507983	0.502362013	0.949878991
0.995064020	0.718370020	0.082598001
0.727959991	0.292360008	0.053608000
0.036660001	0.801935017	0.471347988
0.969429016	0.282561004	0.650336027
0.785803020	0.297360986	0.361416012
0.718005002	0.793914974	0.536898971
0.803578019	0.532500029	0.967233002
0.220693007	0.330058992	0.546338022
0.537025988	0.285284013	0.464028001
0.025760001	0.284188002	0.958833992
0.782616973	0.741994977	0.253580004
0.782599986	0.287178993	0.253805012
0.831580997	0.517220020	0.266993999
0.811013997	0.742662013	0.165114999
0.810645998	0.284276992	0.165248007
0.731314003	0.287108988	0.149053007
0.731629014	0.738744020	0.148879007
0.727975011	0.727491021	0.053435002
0.775538981	0.512326002	0.070058003
0.917200983	0.263884008	0.207130998
0.919425011	0.718838990	0.208831996
0.869678974	0.504787028	0.182766005
0.893598974	0.273110002	0.097172000
0.894915998	0.733521998	0.098453000
0.971751988	0.713989973	0.159824997
0.970457971	0.266285986	0.158059999
0.995540023	0.284963995	0.082580999
0.950664997	0.496540010	0.041636001
0.831843972	0.285598010	0.798741996
0.845982015	0.727421999	0.806532979
0.885836005	0.408078015	0.828612030
0.854839981	0.220912993	0.909094989
0.865508020	0.672892988	0.915547013
0.790425003	0.795883000	0.856818974
0.780279994	0.356810004	0.844901979
0.758065999	0.729409993	0.925189018
0.760855973	0.296371013	0.925611019
0.975611985	0.748749971	0.760508001
0.977420986	0.294597000	0.758709013
0.927473009	0.505554020	0.742859006
0.943624020	0.719612002	0.845561028
0.945447028	0.261889011	0.843685985
0.024698000	0.287117004	0.864987016
0.023644000	0.738161027	0.865644991
0.025776999	0.720421016	0.959411025
0.977827013	0.499642015	0.941637993
0.794125974	0.742233992	0.555948973
0.784983993	0.290482014	0.548627019
0.834284008	0.452544004	0.592347980
0.848622978	0.710418999	0.501788974
0.844124973	0.254785001	0.497357994
0.766237974	0.323886007	0.441747993
0.772937000	0.770983994	0.445042998
0.784682989	0.734349012	0.359690011
0.832302988	0.527409971	0.391400993
0.962302983	0.746416986	0.457872987
0.974261999	0.299717993	0.465335011
0.924546003	0.437929004	0.420020998
0.906854987	0.678496003	0.509503007
0.913700998	0.225445002	0.513760984
0.990541995	0.327423990	0.571699023
0.981782019	0.771063983	0.567960024

0.970528007	0.719919026	0.652661026
0.922753990	0.508845985	0.620454013
0.493591994	0.211478993	0.080874003
0.229500994	0.798309028	0.060238998
0.469855011	0.777936995	0.639285982
0.285362005	0.805594027	0.364948988
0.303997993	0.021537000	0.975728989
0.282409012	0.255003989	0.259754986
0.331248999	0.027128000	0.274327993
0.311877012	0.247819006	0.172310993
0.232397005	0.249476999	0.154580995
0.229407996	0.233866006	0.059749998
0.415569991	0.758562028	0.206644997
0.418190986	0.213661000	0.208557993
0.368357003	0.002434000	0.182115003
0.392383993	0.768586993	0.096819997
0.393635005	0.228942007	0.098043002
0.470616013	0.211358994	0.159050003
0.469487995	0.763630986	0.157775998
0.493887991	0.779976010	0.080890000
0.449099988	0.991401970	0.041131001
0.342316002	0.763212979	0.815903008
0.390767008	0.992161989	0.844026983
0.361844003	0.788906991	0.926144004
0.286321998	0.778881013	0.860053003
0.260107011	0.236098006	0.933194995
0.472525001	0.234972000	0.745643973
0.471989989	0.780119002	0.744175017
0.423234999	0.008319974	0.731096029
0.443787992	0.228560001	0.833292007
0.442604989	0.770035028	0.831632018
0.524904013	0.211353004	0.945527017
0.477685004	0.988026977	0.928342998
0.294380009	0.261774987	0.558640003
0.348724008	0.244008005	0.503682017
0.271762997	0.264759988	0.448170006
0.285008013	0.243520007	0.364080995
0.459621996	0.221387997	0.444321990
0.470353991	0.772810996	0.451750994
0.420296013	0.918551028	0.408583999
0.405561000	0.166507006	0.499015987
0.412274987	0.713522017	0.504357994
0.489841014	0.816766977	0.559207022
0.481070012	0.260861009	0.554825008
0.471132994	0.215330005	0.641242027
0.423375010	0.004680037	0.609959006
0.281987011	0.800176024	0.260522991
0.311037004	0.789317012	0.173021004
0.231645003	0.798003972	0.155077994
0.277173996	0.019116044	0.077147000
0.288875014	0.805845022	0.554778993
0.337058008	0.998149991	0.596369982
0.346776009	0.786675990	0.501955986
0.268842995	0.815607011	0.447182000
0.332525015	0.030441999	0.394392997
0.525442004	0.777329981	0.945927978
0.521763027	0.761283994	0.850220978
0.523603976	0.212668002	0.850670993
0.342938989	0.218176007	0.813017011
0.361651987	0.248704001	0.922783017
0.285908997	0.226713002	0.857465982
0.259647012	0.802253008	0.934099019
0.794564009	0.513738990	0.176513001

0.748929024	0.512771010	0.140065998
0.798192978	0.515103996	0.241218001
0.909452975	0.500123978	0.122491002
0.956896007	0.494222999	0.132209003
0.902980983	0.496847004	0.183480993
0.845614016	0.472458005	0.887467027
0.798846006	0.542446017	0.877183020
0.853278995	0.472357005	0.827169001
0.961390972	0.499680012	0.834609985
0.006309032	0.507583022	0.873314023
0.960264981	0.512830973	0.770748019
0.826144993	0.493447006	0.498430997
0.789848030	0.529133976	0.444061995
0.809159994	0.493456006	0.551787972
0.930525005	0.473224998	0.513522029
0.965906978	0.522077978	0.568704009
0.948705018	0.488602996	0.461245000
0.295024008	0.021003962	0.183587000
0.249651998	0.021857999	0.146318004
0.297982007	0.026128054	0.248052999
0.408243001	0.995748997	0.122093000
0.455680996	0.990474999	0.131764993
0.401674986	0.992546976	0.183015004
0.347397000	0.012170000	0.898961008
0.299721003	0.008848000	0.885949016
0.356900990	0.995526016	0.839981973
0.459652990	0.999136984	0.821637988
0.505051970	0.990990996	0.858913004
0.456560999	0.005979000	0.757138014
0.327558011	0.019216999	0.502053022
0.290668994	0.032623000	0.448018998
0.311291009	0.020199999	0.555970013
0.429008991	0.959999979	0.502537012
0.465353012	0.012261033	0.556590974
0.445551008	0.967121005	0.448828012
0.746013999	0.511146009	0.076361001
0.973950982	0.499278009	0.080123000
0.780858994	0.521125972	0.928225994
0.007666945	0.502174973	0.936452985
0.800477982	0.519975007	0.387151003
0.954626977	0.504969001	0.625091016
0.247476995	0.017818000	0.082858004
0.472548991	0.993752003	0.079462998
0.281300008	0.018649001	0.936684012
0.507357001	0.992334008	0.922523975
0.300794989	0.027061000	0.390783995
0.455132991	0.999678016	0.613752007

****

HTO-HDA

****

HTO-HDA: $(H_2Ti_4O_9)_4(NH_2C_6H_{12}NH_2)_4$

1.0

34.1762657166	0.0000000000	0.0000000000
0.0189009351	3.8782100248	0.0000000000
-4.5555123181	0.0205129344	11.0035548259

O	Ti	H	C	N
36	16	72	24	8

Direct

0.005235000	0.995769024	0.370714009

0.755717993	0.991798997	0.663360000
0.512344003	0.502817988	0.358570993
0.245689005	0.483254999	0.677814007
0.027179999	0.994094014	0.169434994
0.733664989	0.992810011	0.870545030
0.529712975	0.500980020	0.140698999
0.218468994	0.485567987	0.870528996
0.072094999	0.991554976	0.738027990
0.676093996	0.995352030	0.277498990
0.587373018	0.498881012	0.736899018
0.170071006	0.488031000	0.284280986
0.090462998	0.991137028	0.537065029
0.666819990	0.995607018	0.496358991
0.601166010	0.498447001	0.527873993
0.159546003	0.487982988	0.504737020
0.110932000	0.990420997	0.311751992
0.649650991	0.996285021	0.722084999
0.615392029	0.497851998	0.296981007
0.134644002	0.488799006	0.721032977
0.133884996	0.989040017	0.098300003
0.619084001	0.997606993	0.921849012
0.643765986	0.496594995	0.093547001
0.109596997	0.490018010	0.931177974
0.181759000	0.986784995	0.683293998
0.574009001	0.999637008	0.349242002
0.693310976	0.494435012	0.675144970
0.067359999	0.492442995	0.360287994
0.593398988	0.998678982	0.128061995
0.155259997	0.987944007	0.886659026
0.087200001	0.491189986	0.144178003
0.669667006	0.495445997	0.886937976
0.702830970	0.993892014	0.079444997
0.049343999	0.992733002	0.951673985
0.194061995	0.485850990	0.082400002
0.559961975	0.500105023	0.935769022
0.057666998	0.993059993	0.367752999
0.703738987	0.994059980	0.665823996
0.565011978	0.500056982	0.362264007
0.193215996	0.486259013	0.674350977
0.070308998	0.991966009	0.124082997
0.688561976	0.994652987	0.906850994
0.575685024	0.499392986	0.110955000
0.173519000	0.487170011	0.909362972
0.125156000	0.989220023	0.692409992
0.632411003	0.997093976	0.341939986
0.634845018	0.496937007	0.683233976
0.126096994	0.489771008	0.349559009
0.649626017	0.996419013	0.111236997
0.094754003	0.990620971	0.904321015
0.141792998	0.488649011	0.118421003
0.612075984	0.497898996	0.901314974
0.491818994	0.784196019	0.267468989
0.536725998	0.786584973	0.834335029
0.265740991	0.201600000	0.769219995
0.210317001	0.982333004	0.295897990
0.401192993	0.226546004	0.888248026
0.402287006	0.773747981	0.888879001
0.328262001	0.219999999	0.719619989
0.329461992	0.766535997	0.719119012
0.337453008	0.758337975	0.938193023
0.336349010	0.214454994	0.938780010
0.492821991	0.007378000	0.145000994
0.266337991	0.761910021	0.769199014

0.398494989	0.230591998	0.674457014
0.398988992	0.775915980	0.673985004
0.468603998	0.229460999	0.855201006
0.468964994	0.777938008	0.855629027
0.471311986	0.775924027	0.645485997
0.471262008	0.231624007	0.645582020
0.536714971	0.215688005	0.834383011
0.543649018	0.000945000	0.719092011
0.356296003	0.217852995	0.356265008
0.357111990	0.763176978	0.356242001
0.287844986	0.213210002	0.169830993
0.288610995	0.761080027	0.170123994
0.282674998	0.756013989	0.375398993
0.218186006	0.199017003	0.183284000
0.356602997	0.767795026	0.144611001
0.427891999	0.225674003	0.316347986
0.428795010	0.771920979	0.316356003
0.421420008	0.774222016	0.097759001
0.264755994	0.979445994	0.891927004
0.491423994	0.224555999	0.267922014
0.282189995	0.211596996	0.375768989
0.218476996	0.769625008	0.183029994
0.355726004	0.220838994	0.144511998
0.420702010	0.230777994	0.097979002
0.986603022	0.714134991	0.267318010
0.785338998	0.490393996	0.871396005
0.982219994	0.719051003	0.755270004
0.961561024	0.723191023	0.525852978
0.038639002	0.708253980	0.651475012
0.996424973	0.497595012	0.157309994
0.024878001	0.493434995	0.518400013
0.982492030	0.267740995	0.755122006
0.961636007	0.268308014	0.525416970
0.038690001	0.277617007	0.651472986
0.921809018	0.724999011	0.284651995
0.922115982	0.272646993	0.285019994
0.923657000	0.269923002	0.075502001
0.986701012	0.279359996	0.267107010
0.923533976	0.727585971	0.075456999
0.919435978	0.718302011	0.834713995
0.843424022	0.718358994	0.680335999
0.855544984	0.717316985	0.902661026
0.904797971	0.720817983	0.610477984
0.781131029	0.712704003	0.744145989
0.714923024	0.491710007	0.303021014
0.919794023	0.264946014	0.834501982
0.843775988	0.265197009	0.680149019
0.855804026	0.261482000	0.902381003
0.781271994	0.268913001	0.744212985
0.905077994	0.267601013	0.610144019
0.856647015	0.724197984	0.326995999
0.857133985	0.270191014	0.327165991
0.784588993	0.721562982	0.155267000
0.785099030	0.270184010	0.155246004
0.788215995	0.265228987	0.371206999
0.722383976	0.279621005	0.187702000
0.850983024	0.271293998	0.109779000
0.787675023	0.721776009	0.371190995
0.722057998	0.705348015	0.187573001
0.850610018	0.725983024	0.109747000
0.341679990	0.993317008	0.777907014
0.325087011	0.986698985	0.879904985
0.389858007	0.999282002	0.828813016

0.457982004	0.003538000	0.794748008
0.480033994	0.003337000	0.705283999
0.409767002	0.002602000	0.734076977
0.298238009	0.987296999	0.230840996
0.274441987	0.984508991	0.315811008
0.346327990	0.990624011	0.296146005
0.415715009	0.998560011	0.257701010
0.432738990	0.002278000	0.156572998
0.367565989	0.994445026	0.204606995
0.967302978	0.493786991	0.698704004
0.975934982	0.494877994	0.581664979
0.910510004	0.498744994	0.224776000
0.932111979	0.498461992	0.134896994
0.857470989	0.491414994	0.737887025
0.842975020	0.489973009	0.844511986
0.905820012	0.492002994	0.776784003
0.919300020	0.493647009	0.667757988
0.796944976	0.495732009	0.215127006
0.778217971	0.493705004	0.311906010
0.844772995	0.497177005	0.267682999
0.862702012	0.498436004	0.169027999
0.278782010	0.981728971	0.829575002
0.526221991	0.001590000	0.773145974
0.228515998	0.983803988	0.243944004
0.478893995	0.004809000	0.207641006
0.021500001	0.493580014	0.600881994
0.977496982	0.497245997	0.206545994
0.796473026	0.490303010	0.802506983
0.732070982	0.492385000	0.248456001

****

References

[1] H. Izawa, S. Kikkawa, M. Koizumi, *J. Phys. Chem.* **1982**, *86*, 5023–5026.

[2] G. Kresse, J. Furthmüller, *Comput. Mater. Sci.* **1996**, *6*, 15–50.

[3] G. Kresse, J. Furthmüller, *Phys. Rev. B* **1996**, *54*, 11169–11186.

[4] J. P. Perdew, K. Burke, M. Ernzerhof, *Phys. Rev. Lett.* **1996**, *77*, 3865–3868.

[5] P. E. Blöchl, *Phys. Rev. B* **1994**, *50*, 17953–17979.

[6] J. D. Pack, H. J. Monkhorst, *Phys. Rev. B* **1977**, *16*, 1748–1749.

[7] S. Grimme, J. Antony, S. Ehrlich, H. Krieg, *J. Chem. Phys.* **2010**, *132*, 154104.

[8] I. A. Rodionov, E. A. Maksimova, A. Y. Pozhidaev, S. A. Kurnosenko, O. I. Silyukov, I. A. Zvereva, *Front. Chem.* **2019**, *7*, 863.

[9] S. A. Kurnosenko, V. V. Voytovich, O. I. Silyukov, I. A. Minich, E. N. Malygina, I. A. Zvereva, *Ceram. Int.* **2022**, *48*, 7240–7252.

[10] S. Tahara, T. Ichikawa, G. Kajiwara, Y. Sugahara, *Chem. Mater.* **2007**, *19*, 2352–2358.

[11] A. M. Amorim da Costa, M. P. M. Marques, L. A. E. Batista de Carvalho, *Vib. Spectrosc.* **2002**, *29*, 61–67.

[12] W. Choi, H.-C. Shin, J. M. Kim, J.-Y. Choi, W.-S. Yoon, *J. Electrochem. Sci. Technol.* **2020**, *11*, 1–13.

[13] N. Shpigel, M. D. Levi, D. Aurbach, *Energy Storage Mater.* **2019**, *21*, 399–413.

[14] I. Reviakine, D. Johannsmann, R. P. Richter, *Anal. Chem.* **2011**, *83*, 8838–8848.

[15] A. D. Easley, T. Ma, C. I. Eneh, J. Yun, R. M. Thakur, J. L. Lutkenhaus, *J. Polym. Sci.* **2022**, *60*, 1090–1107.
